# Finite element analysis for assessing the flexural performance of CFRP-strengthened composite slabs without overextending reinforcing bars

**DOI:** 10.1371/journal.pone.0356379

**Published:** 2026-08-14

**Authors:** Lu Qi, Qi Lai, Junyun Liao, Haiping Luo, Xiaojuan Yu, Xi Chen, Qun Mai, Xiaosan Yin

**Affiliations:** 1 Jiangxi Zhongmei Engineering Group Company Limited, Nanchang, Jiangxi, China; 2 Engineering Quality Management Service Center, Ganzhou, Jiangxi, China; 3 School of Intelligent Construction and Civil Engineering, Zhongyuan University of Technology, Zhengzhou, Henan, China; University of Vigo, SPAIN

## Abstract

To address the inherent limitations in design, construction, and quality control associated with the use of overextending reinforcing bars in traditional precast composite slabs, this paper proposes a novel precast composite slab configuration that eliminates such reinforcement protrusions. The joints of the proposed slabs are strengthened using Carbon Fiber Reinforced Polymer (CFRP), and the key construction techniques of this strengthening system are systematically presented. A refined three-dimensional finite element model was developed using the ABAQUS platform to investigate the effects of critical parameters—including the number of CFRP layers, CFRP thickness, and bonding length—on the yield load and deformation behavior of the strengthened composite slabs. The numerical results demonstrate that CFRP effectively restores the stress transfer path in the joint region through a bridging mechanism, thereby improving the load-bearing capacity of the composite slabs. However, when the bonding length exceeds the stress transfer length of the reinforcing bars embedded in concrete, further increases in either the number of CFRP layers or the bonding length yield diminishing returns in terms of load-bearing enhancement. Based on the parametric analysis, a design methodology for CFRP strengthening is proposed, centered on the principles of equal-strength force transmission and effective bond transfer. This approach provides a theoretical foundation for the practical application and wider adoption of CFRP-strengthened composite slabs without overextending reinforcing bars.

## Introduction

With the continuous acceleration of urbanization and the in-depth advancement of construction industrialization, precast concrete structures have been widely adopted in high-rise buildings, industrial plants, and bridge engineering, owing to their prominent advantages such as high construction efficiency, quality controllability, and reduced environmental impact on-site. As a critical horizontal load-bearing component in precast concrete structural systems, the precast composite slab effectively integrates the precision of factory prefabrication with the structural integrity of cast-in-situ concrete through the collaborative interaction between the precast bottom slab and the cast-in-situ concrete layer. During the construction phase, the precast bottom slab serves directly as permanent formwork for the subsequent concrete casting, which not only significantly reduces on-site formwork workload but also shortens construction periods and enhances overall construction efficiency. In the service stage, the precast layer and the cast-in-situ layer work together as a monolithic unit, resulting in structural performance comparable to that of fully cast-in-place concrete slabs. This configuration simultaneously offers the combined benefits of factory-controlled production quality and a high degree of construction industrialization [1,2].

Despite these advantages, the conventional design approach involving overextending reinforcing bars in precast composite slabs presents persistent challenges throughout the entire process of design, production, and installation. In the design phase, the need to accommodate overextending bars substantially increases design complexity and detailing requirements. During production, the presence of protruding bars restricts automation levels and leads to higher mold fabrication costs. Furthermore, during handling, stacking, and transportation of precast components, these exposed bars are susceptible to bending, breakage, or corrosion, introducing potential quality risks and increasing repair costs. At the installation stage, conflicts frequently arise between bars extending from adjacent slab sides, requiring on-site adjustments and additional formwork for cast-in-place strips, which further complicates the construction process and undermines efficiency [3,4].

To address these issues, composite slabs without overextending reinforcing bars have been proposed as an alternative configuration that can effectively avoid many of the aforementioned drawbacks. Recent studies have explored various alternative joint concepts to improve the performance of such systems. For instance, Zhang et al. [2] investigated the flexural performance of novel wet joints with sleeve connections, while Wang et al. [4] examined the mechanical properties of a new type of composite floor slab with a split joint. Lou et al. [5] also studied the mechanical behavior of a reinforced concrete composite slab with a joint. However, a critical challenge persists: the force transfer mechanism at the slab-to-slab joints remains a weak link that significantly influences the overall structural performance. In designs without overextending bars, the discontinuity of both concrete and longitudinal reinforcement across the joint prevents effective tensile stress transfer, limiting the load-bearing contribution of the precast bottom slab. Therefore, exploring high-performance joint solutions is essential for ensuring the successful application of such composite slabs [5].

In this context, CFRP offers unique advantages, including a high strength-to-weight ratio, excellent corrosion resistance, and convenient construction procedures, making it a mainstream material in the field of concrete structure strengthening [6]. Extensive research [7] has demonstrated that CFRP can significantly enhance the flexural and shear capacities as well as the deformation ability of reinforced concrete beams, columns, and other structural components. When CFRP is bonded at the joints of precast bottom slabs, it effectively provides localized strengthening, thereby restoring or even improving the overall performance of the composite slab system. Beyond structural strengthening, related studies on bond mechanics provide valuable insights. For example, research on bonded interfaces in stone-clad façades has demonstrated the effectiveness of finite element modeling with element deletion techniques to simulate crack initiation and propagation [8], and has shown how mechanical anchorage and material properties like fiber dosage influence shear bond strength [9]. These findings inform the numerical strategies for simulating the CFRP-concrete interface and the role of anchorage in this study.

**Fig 1:**
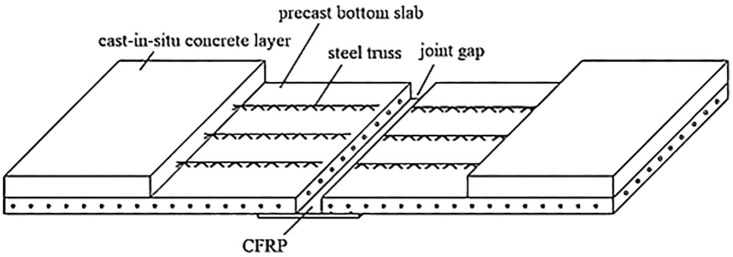
Schematic diagram.

Based on the design concept of composite slabs without overextending reinforcing bars and leveraging the high-strength, lightweight properties of carbon fiber, this paper proposes a novel structural configuration: CFRP-strengthened composite slabs without overextending reinforcing bars. This innovative approach aims to overcome the limitations associated with conventional joint detailing while maintaining construction efficiency and structural reliability. The proposed configuration is schematically illustrated in Fig 1.

Existing research on composite slabs has predominantly centered on the mechanical performance of conventional configurations featuring overextending reinforcing bars. For instance, Shen et al. [10] systematically investigated the influence of interface roughness on the overall flexural behavior through experimental studies. Li et al. [2] developed a calculation model for the flexural capacity of ribbed composite slabs and validated the enhancing effect of steel trusses. In the domain of CFRP strengthening, Wang et al. [11] confirmed the effectiveness of CFRP in controlling crack propagation in reinforced concrete beams through four-point bending tests, while Wang et al. [12] conducted a systematic investigation into the bond-slip constitutive relationship at the CFRP-concrete interface. Furthermore, advanced numerical simulations have been employed to assess structural performance under various conditions, including fatigue and torsion [13,14], while innovative strengthening techniques like the side near-surface mounted method have shown significant improvements in flexural performance [15]. Despite these valuable contributions, a significant research gap remains. The existing findings cannot be directly applied to the flexural design and analysis of CFRP-strengthened composite slabs without overextending reinforcing bars, owing to the fundamentally different force transfer mechanism at the slab joints where both concrete and reinforcement are intentionally discontinued.

To address this research gap, the present study focuses on the flexural performance of such composite slabs by establishing a refined three-dimensional solid finite element model using the ABAQUS platform. The major challenges include accurately simulating the discontinuous reinforcement, the joint gap and capturing the progressive failure of the system. Our original contributions to overcome these challenges are: (1) the proposition of a novel CFRP-strengthened composite slab system that eliminates protruding bars, significantly enhancing constructability; (2) the development of a validated FE model that captures the unique bridging mechanism of CFRP across the discontinuous joint; (3) the identification of a saturation effect for CFRP layers and bonding length, providing an optimal design threshold; and (4) the proposal of a practical design methodology based on equal-strength and effective bond transfer principles. Through material nonlinear analysis and parametric simulations, the effects of key variables—including the number of CFRP layers, CFRP thickness, and bonding length—on the yield load and deformation behavior are systematically investigated. The findings are intended to provide a theoretical basis and optimized design recommendations for practical engineering applications. The proposed system is particularly suited for precast concrete construction where rapid assembly, reduced logistical complexity, and enhanced quality control are paramount, offering a more efficient and reliable alternative to traditional composite slab systems.

## Construction techniques for CFRP-strengthened composite slabs without overextending reinforcing bars

### Substrate construction and formwork erection of the composite slab

The CFRP strengthening of composite slabs without overextending reinforcing bars fundamentally depends on the quality of the substrate, which is formed jointly by the precast bottom slab and the cast-in-situ concrete layer. The construction process commences with the precise installation of the precast bottom slabs. During hoisting and positioning, precision instruments such as laser alignment devices are employed to ensure that the joint width between adjacent precast slabs does not exceed 2 mm, and that the elevation error of the slab surface is strictly controlled within ±3 mm [16].

The formwork support system is required to be rigorously designed. The spacing of steel supports should typically not exceed 900 mm. Primary beams are preferably fabricated from 100 mm × 100 mm square steel tubing, while secondary beams may consist of 50 mm × 100 mm wooden battens, with their spacing controlled at 250 mm. To meet structural camber requirements, the free end length of the top support should be limited to no more than 300 mm, and a camber of 0.1% to 0.3% of the span length should be applied during formwork erection.

Following formwork installation, the cast-in-situ concrete layer is poured. The pouring sequence should proceed from the center of the slab outward in a radiating manner to ensure uniform distribution and minimize differential settlement. Secondary vibration is performed at the interface between the precast bottom slab and the cast-in-situ concrete layer to achieve a dense and well-bonded contact between the existing and fresh concrete. Upon completion of pouring, immediate covering with plastic film and moist curing for no less than seven days are required. The ambient temperature during curing should preferably be maintained at 20 ± 5 °C to ensure proper hydration and strength development. Subsequent CFRP strengthening operations may proceed only after the concrete has attained the specified design strength, as verified through appropriate testing.

### Substrate preparation before strengthening and CFRP bonding

After the cast-in-situ concrete layer has hardened and attained the required strength, the core construction phase for CFRP strengthening commences. The CFRP strengthening layout is applied to the underside of the slab, directly bridging the joint between adjacent precast bottom slabs. The overall quality of the strengthening work is largely contingent upon meticulous substrate preparation. The process begins with the joint area of the precast bottom slab, where CFRP is to be bonded, being thoroughly roughened until solid aggregate is exposed, thereby achieving a surface profile with an average valley-to-peak height of no less than 4 mm. The flatness of the concrete substrate must satisfy the requirement that the maximum gap beneath a 2 m straightedge does not exceed 5 mm. For any existing cracks wider than 0.2 mm, low-pressure epoxy resin injection (at 0.2–0.4MPa) should be employed for effective sealing. Surface defects such as honeycombing or spalling must be repaired using polymer mortar with a compressive strength not lower than the original design grade, ensuring that the bond strength between the repair material and the substrate is at least 2.5MPa. Finally, the substrate must be rendered dry and clean, with a moisture content not exceeding 4%. This is achieved through a combination of air blowing and acetone wiping, continuing until a white cloth wiped across the surface shows no signs of contamination.

Upon satisfactory completion of substrate preparation, the CFRP bonding procedure is executed as follows. First, an epoxy primer is applied evenly using a roller, with the thickness controlled within 0.4 ± 0.1 mm. Once the primer has become touch-dry, any depressions or irregularities at corners are leveled using epoxy filler [17]. Subsequently, epoxy saturant resin is spread uniformly onto the bonding surface at an approximate application rate of 500g/m². The pre-cut CFRP sheets are then carefully laid along the direction of the span’s primary tensile stress, with appropriate tension applied during placement to ensure full contact and fiber alignment. A dedicated rubber roller is used to apply firm pressure repeatedly for at least three passes, systematically expelling entrapped air bubbles until resin exudes uniformly from the edges of the fabric, thereby confirming complete impregnation and intimate bonding between the CFRP and the concrete substrate.

### Quality control, detail treatment, and safety & environmental protection measures

To ensure the long-term effectiveness and reliability of CFRP strengthening, rigorous quality control measures must be implemented throughout the construction process, with particular attention given to critical details. The primary inspection items include the following [18]: detection of bonding voids using the tap test method supplemented by infrared imaging, with acceptance criteria requiring that any single void area does not exceed 1000 mm² and that the total void area ratio remains within 5%; verification of CFRP sheet positioning using a total station, ensuring that axis deviation is limited to no more than 5 mm; measurement of adhesive layer thickness employing an ultrasonic thickness gauge, with allowable deviation controlled within 0.5 ± 0.1 mm; and finally, validation of the strengthened structure’s load-bearing capacity through static load testing, confirming that it can withstand at least 1.25 times the design load without exhibiting signs of distress or failure.

With regard to safety and environmental protection considerations, wet cutting methods should be employed during construction operations, supplemented by mobile dust collectors, to effectively control on-site dust concentration and minimize airborne particulate emissions. For work conducted at height, suspended working platforms with a load capacity of not less than 2. kN/m² must be utilized, ensuring compliance with occupational safety standards and providing a secure working environment. These measures collectively contribute to achieving comprehensive safety management and environmentally responsible construction practices throughout the entire CFRP strengthening process.

## Establishment of the finite element model

To achieve a refined numerical simulation of the flexural behavior of CFRP-strengthened composite slabs without overextending reinforcing bars, a three-dimensional finite element model that accurately captures the mechanical response of such structural components was developed using the ABAQUS platform.

### Geometric model and component assembly

During the modeling process, to accurately simulate the mechanical behavior at the composite interface of the slab without overextending reinforcement, the precast bottom slab and the cast-in-situ concrete layer were created as separate solid entities, with interfacial contact properties explicitly defined between them. The steel trusses embedded within the precast bottom slab were simulated using truss elements (T3D2) and integrated with the surrounding concrete through an “embedded” constraint, thereby ensuring full compatibility of deformation. The CFRP reinforcement was modeled using shell elements (S4R). Given its significantly smaller thickness relative to its planar dimensions, the CFRP was precisely positioned and configured to conform to the tension zone on the bottom surface of the cast-in-situ concrete layer. All components were accurately assembled through Boolean operations and meticulous positional adjustments, ensuring initial geometric conformity between the CFRP and the concrete substrate.The concrete solids, including both the precast bottom slab and the cast-in-situ layer, were discretized using eight-node linear reduced-integration hexahedral elements (C3D8R). This element type is particularly suitable for large deformation and contact analyses, as it effectively suppresses hourglassing while maintaining computational efficiency [19]. The CFRP sheets were modeled using four-node reduced-integration shell elements (S4R), which adequately capture the membrane and bending behavior of the thin composite material.

**Fig 2:**
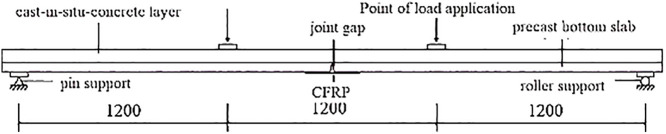
Schematic diagram of loading.

In terms of mechanical response, the behavior of the CFRP-strengthened composite slab without overextending reinforcing bars in the direction parallel to the short edges is consistent with that of conventional composite slabs. However, due to the specific slab-to-slab connection detail, distinct mechanical characteristics emerge in the direction perpendicular to the joint. To investigate this directional difference, a finite element simulation employing a four-point bending configuration was applied to the slab, creating a constant-moment region at the mid-span where the slab joint and CFRP reinforcement are located. In this configuration, two symmetric vertical loads are applied to generate a pure bending zone, allowing the flexural behavior of the CFRP-strengthened joint to be evaluated without the interference of shear forces. The numerical model comprehensively incorporates the CFRP reinforcement, precast bottom slab, longitudinal reinforcement, cast-in-situ concrete layer, and steel trusses. The slab geometry is defined by a clear span of 3600 mm, a width of 1000 mm, a precast bottom slab thickness of 60 mm, and a cast-in-situ concrete layer thickness of 100 mm. The joint gap between adjacent precast bottom slabs was set to 1 mm to reflect the actual construction condition. A schematic representation of the loading configuration is provided in Fig 2.

### Material constitutive models and parameters

#### Concrete.

Both the precast bottom slab and the cast-in-situ concrete layer were modeled using the Concrete Damaged Plasticity (CDP) model available in ABAQUS. This constitutive model effectively simulates the mechanical behavior of concrete under uniaxial and multiaxial loading conditions, capturing key phenomena such as compressive crushing and tensile cracking. The uniaxial compressive and tensile stress-strain relationships for the two types of concrete were separately defined in the model based on established formulations [2,14].

The uniaxial tensile stress-strain curve of concrete can be calculated using the following formula:


$$\begin{array}{c} \sigma =\text{(}1-d_{\text{t}})E_{\text{c}}\varepsilon \end{array}$$ (1)

where $E_{\text{c}}$ is the elastic modulus; $d_{\text{t}}$ is the damage evolution coefficient of concrete under uniaxial tension, which is calculated as follows:


$$\begin{array}{c} d_{\text{t}}=\left\{ \begin{array}{c} @l@1-\rho_{\text{t}}[1.2-0.2x^{\text{5}}]\begin{array}{cc} \begin{array}{cc} & x\leq 1 \end{array} & \end{array}\\ 1-\frac{\rho_{\text{t}}}{\alpha_{\text{t}}(x-1{)}^{1.7}+x}\begin{array}{cc} & \;x>1 \end{array} \end{array}\right. \end{array}$$ (2)

where α is the reference value for the descending branch of the uniaxial tensile stress-strain curve of concrete. The values of the relevant parameters are provided below:


$$\begin{array}{c} \left\{ \begin{array}{c} @l@x=\frac{\varepsilon }{\varepsilon_{\text{t,r}}}\\ \rho_{\text{t}}=\frac{f_{\text{t,r}}}{E_{\text{c}}\varepsilon_{\text{t,r}}} \end{array}\right. \end{array}$$ (3)

where $f_{\text{t,r}}$ is the representative value of the uniaxial tensile strength of concrete, and $\varepsilon_{\text{t,r}}$ is the peak tensile strain corresponding to that representative value.

The uniaxial compressive stress-strain curve of concrete can be calculated using the following formula:


$$\begin{array}{c} \sigma =\text{(}1-d_{\text{t}})E_{\text{c}}\varepsilon \end{array}$$ (4)

Here, $d_{\text{t}}$ is the damage evolution coefficient of concrete under uniaxial compression, calculated as follows:


$$\begin{array}{c} d_{\text{c}}=\left\{ \begin{array}{c} @l@1-\frac{\rho_{\text{c}}n}{n-1+x^{n}}\begin{array}{cc} \begin{array}{cc} & x\leq 1 \end{array} & \end{array}\\ 1-\frac{\rho_{\text{c}}}{\alpha_{\text{c}}(x-1{)}^{\text{2}}+x}\begin{array}{cc} & x>1 \end{array} \end{array}\right. \end{array}$$ (5)

where $\alpha_{\text{c}}$ is the reference value for the descending branch of the uniaxial compressive stress-strain curve of concrete. The values of the relevant parameters are specified as follows:


$$\begin{array}{c} \left\{ \begin{array}{c} @l@x=\frac{\varepsilon }{\varepsilon_{\text{c,r}}}\\ \rho_{\text{t}}=\frac{f_{\text{c,r}}}{E_{\text{c}}\varepsilon_{\text{c,r}}}\\ n=\frac{E_{\text{c}}\varepsilon_{\text{c,r}}}{E_{\text{c}}\varepsilon_{\text{c,r}}-f_{\text{c,r}}} \end{array}\right. \end{array}$$ (6)

where $f_{\text{c,r}}$ is the representative value of the uniaxial compressive strength of concrete, and $\varepsilon_{\text{c,r}}$ is the peak compressive strain corresponding to that representative value.

The failure criterion of the CDP model is based on the Lubliner-Lee yield criterion, and a non-associated plastic flow rule is adopted [20,21]. The main parameters are as follows: the dilation angle was taken as 30°, the eccentricity parameter as 0.1, and the ratio of biaxial compressive strength to uniaxial compressive strength was assigned a value of 1.16. To enhance computational convergence, a viscosity coefficient of 0.001 was introduced. The compressive strength of the precast slab concrete was 35.6 MPa, while that of the cast-in-situ concrete was 39.45 MPa. Poisson’s ratio was taken as 0.2 for both materials, and the elastic modulus was determined in accordance with the Chinese standard *General Code for Concrete Structures* (GB 55008−2021).

#### Steel bars.

Plain round steel bars of 8 mm diameter, grade HPB300, were used as reinforcement in both the precast bottom slab and the cast-in-situ concrete layer. Their yield strength was 345 MPa, tensile strength 450 MPa, and elastic modulus 2.0 × 10⁵ MPa. In the precast bottom slab, the longitudinal tension reinforcement was spaced at 150 mm, while the distribution reinforcement was placed at a spacing of 250 mm.

In ABAQUS, steel is modeled by default using the Von Mises yield criterion with an associated plastic flow rule. All steel bar was modeled as an ideal elastoplastic material using a bilinear constitutive model [22], which adequately captures both the elastic stiffness and the post-yield plateau behavior. The stress-strain constitutive relationship of the steel under monotonic loading can be expressed by the following equation:


$$\begin{array}{c} \sigma_{\text{s}}=\left\{ \begin{array}{c} @l@E_{\text{s}}\varepsilon_{\text{s}}\begin{array}{cc} & \varepsilon_{\text{s}}\leq \varepsilon_{\text{y}} \end{array}\\ f_{\text{y,r}}\begin{array}{cc} & \varepsilon_{\text{y}}<\varepsilon_{\text{s}}\leq \varepsilon_{\text{uy}} \end{array}\\ f_{\text{y,r}}+k(\varepsilon_{\text{s}}-\varepsilon_{\text{uy}})\begin{array}{cc} & \varepsilon_{\text{y}}<\varepsilon_{\text{s}}\leq \varepsilon_{\text{uy}} \end{array}\\ 0\begin{array}{cc} & \varepsilon_{\text{s}}>\varepsilon_{\text{u}} \end{array} \end{array}\right. \end{array}$$ (7)

where $E_{\text{s}}$ is the elastic modulus of the steel reinforcement; $\sigma_{\text{s}}$ is the stress of the steel reinforcement; $\varepsilon_{\text{s}}$ is the strain of the steel reinforcement; $f_{\text{y,r}}$ is the representative value of the yield strength of the steel reinforcement; $\varepsilon_{\text{y}}$ is the yield strain of the steel reinforcement corresponding to $f_{\text{y,r}}$; $\varepsilon_{\text{uy}}$ is the strain at the onset of strain hardening of the steel reinforcement; $\varepsilon_{\text{u}}$ is the peak strain of the steel reinforcement; k is the slope of the strain hardening branch of the steel reinforcement.

#### CFRP.

CFRP exhibits linear elastic behavior up to brittle fracture without any plastic deformation capacity. Therefore, its constitutive relationship is simplified as a linear elastic model in the finite element analysis, as commonly adopted in the literature for FRP-strengthened concrete members under various loading conditions [14,23]. The uniaxial tensile stress-strain relationship of CFRP can be expressed as:


$$\begin{array}{c} \sigma_{\text{CFRP}}=E_{\text{CFRP}}\varepsilon_{\text{CFRP}},0\leq \varepsilon_{\text{CFRP}}\leq \varepsilon_{\text{ult}} \end{array}$$ (8)

where $\sigma_{\text{CFRP}}$ and $\varepsilon_{\text{CFRP}}$ are the tensile stress and strain of the CFRP, respectively; $E_{\text{CFRP}}$ is the elastic modulus; and $\varepsilon_{\text{ult}}$ is the ultimate tensile strain at failure. Once the tensile strain exceeds $\varepsilon_{\text{ult}}$, the material is considered to have failed.

To simulate the sudden brittle fracture of CFRP, a maximum principal stress failure criterion is incorporated into the material definition. Failure is assumed to occur when the maximum principal stress in any element reaches the specified ultimate tensile strength. Upon satisfying this criterion, the corresponding element is deactivated (removed from the calculation) in the Abaqus solver, thereby realistically capturing the rupture behavior of the composite material under tension. This element-deactivation technique has been successfully used in previous studies to model the progressive failure of CFRP-strengthened concrete structures [14, 24].

The single-layer CFRP adopted in this study had a tensile strength of 4148 MPa, an elastic modulus of 225 GPa, an ultimate tensile strain of approximately 0.0184, an interlayer shear strength of 52 MPa, and a Poisson’s ratio of 0.3 [12].These parameters are used directly in the linear elastic constitutive model to define the mechanical response of CFRP in the finite element simulations.

#### Interface simulation.

To accurately simulate the bond-slip behavior and potential interfacial debonding failure between the CFRP and the concrete substrate, three-dimensional cohesive elements (COH3D8) were employed [22,25]. These elements are capable of effectively describing the progressive debonding process of the interface under combined tensile and shear actions [14,24]. Their constitutive behavior follows a traction-separation law [12], which consists of two stages: a linear elastic stage governed by an initial stiffness matrix, followed by a damage initiation and evolution stage based on an energy-based fracture criterion [26].

The linear elastic behavior is defined by a stiffness matrix relating the nominal traction stresses ***t***={*t*_n_,*t*_s_,*t*_t_} to the separations *δ*={*δ*_n_,*δ*_s_,*δ*_t_}, where the subscripts *n*, *s*, and *t* denote the normal and two shear directions, respectively. For an uncoupled response, the elastic constitutive relation is given by:


$$\begin{array}{c} t=[\begin{array}{ccc} @ccc@K_{\text{nn}} & 0 & 0\\ 0 & K_{\text{ss}} & 0\\ 0 & 0 & K_{\text{tt}} \end{array}) \end{array}$$ (9)

where *K*_nn_ = 1.5 × 10^3^ MPa/mm is the normal stiffness, and *Kss* = *K*_tt_ = 5.0 × 103 MPa/mm are the tangential stiffnesses. These stiffness values are selected based on typical epoxy adhesive properties and have been adopted in previous numerical studies on CFRP-strengthened concrete members [12,14].

Damage initiation is governed by the quadratic stress criterion [27], which assumes that damage begins when the following condition is satisfied:


$$\begin{array}{c} {\left(\frac{\langle t_{\text{n}}\rangle }{t_{\text{n}}^{\text{0}}}\right)}^{\text{2}}+{\left(\frac{\langle t_{\text{s}}\rangle }{t_{\text{s}}^{\text{0}}}\right)}^{\text{2}}+{\left(\frac{\langle t_{\text{t}}\rangle }{t_{\text{t}}^{\text{0}}}\right)}^{\text{2}}=1 \end{array}$$ (10)

where $t_{\text{n}}^{\text{0}}$ =4.0 MPa is the normal interfacial tensile strength (bond strength to concrete), and $t_{\text{s}}^{\text{0}}$ = $t_{\text{t}}^{\text{0}}$ =6.0 MPa are the shear strengths. The Macaulay brackets ⟨⟩ indicate that pure compression does not initiate damage. These strength values are consistent with the epoxy resin properties reported by the manufacturer and have been used in similar finite element studies [14,22]. The shear strength is estimated as 1.5 times the bond strength based on typical epoxy adhesive behavior [12].

After damage initiation, the damage evolution follows a mixed-mode energy-based fracture criterion described by the Benzeggagh-Kenane law [28].

The total mixed-mode fracture energy *G*^C^is expressed as:


$$\begin{array}{c} G^{\text{C}}=G_{\text{n}}^{\text{C}}+(G_{\text{s}}^{\text{C}}-G_{\text{n}}^{\text{C}})(\frac{G_{\text{s}}+G_{\text{t}}}{G_{\text{n}}+G_{\text{s}}+G_{\text{t}}}{)}^{\eta } \end{array}$$ (11)

where $G_{\text{n}}^{\text{C}}$ =0.2 N/mm is the mode-I (normal) fracture energy, $G_{\text{s}}^{\text{C}}$ = $G_{\text{t}}^{\text{C}}$ =1.2N/mm is the mode-II fracture energy, and η = 1.45*η* = 1.45 is the BK exponent. The mixed-mode fracture energy is taken as $G^{\text{C}}$ =1.2 N/mm for practical implementation. Damage evolution is assumed to be linear, and the scalar damage variable *D* increases from 0 at initiation to 1 at complete failure, following a displacement-based evolution law [14,24].

Within the ABAQUS environment, the cohesive elements (COH3D8) are inserted as a zero-thickness layer between the CFRP and the concrete substrate. The surfaces of the concrete and the CFRP are each tied to the corresponding faces of the cohesive elements using tie constraints in the Interaction module. Specifically, the master surfaces are assigned to the concrete and CFRP faces, while the slave surfaces are the cohesive element faces. This configuration ensures perfect displacement compatibility and proper stress transfer across the bonded interface prior to damage, and allows the progressive debonding process to be captured once the damage initiation criterion is met. This tie-constraint approach has been validated in previous studies on CFRP-strengthened concrete structures [14,24].

### Interactions, boundary conditions, and loading

Within the Interaction module of ABAQUS, the connections between different components were carefully defined to accurately represent the physical interactions within the composite slab system. The cohesive element layer situated between the CFRP and the concrete substrate, as well as the cohesive contact surface between the precast bottom slab and the cast-in-situ concrete layer, were both connected to their respective host parts using tie constraints. This approach ensures full continuity of displacement and stress transfer across these bonded interfaces, effectively modeling the composite action prior to any potential debonding.

The steel trusses embedded within the precast bottom slab were incorporated using the embedded constraint available in ABAQUS. This constraint automatically ties the translational degrees of freedom of the embedded truss elements to those of the surrounding host concrete elements, thereby ensuring perfect bond and kinematic compatibility between the reinforcement and the concrete matrix.

Surface-to-surface contact interactions were defined between the loading plates and the top surface of the composite slab. The normal behavior was modeled as “hard” contact, which minimizes penetration while allowing separation after contact. The tangential behavior was simulated using the Coulomb friction model, with a friction coefficient of 0.3 assigned to characterize the frictional resistance at the contact interface.

Boundary conditions were established in accordance with a typical simply supported test configuration. At one support end of the composite slab, both the vertical displacement (U2 = 0) and the in-plane horizontal displacement (*U*_1_ = 0) were constrained to simulate a hinged support condition. At the opposite end, only the vertical displacement (*U*_2_ = 0) was restrained, thereby creating a roller support condition that releases any restraint stresses that might otherwise develop due to factors such as thermal expansion or shrinkage along the span direction.

To enable stable and comprehensive tracking of the structural response beyond the peak load and throughout the complete failure process, a displacement-controlled loading scheme was adopted. Vertical displacements were progressively imposed at the loading locations indicated in Fig 2, allowing for the observation of post-peak softening behavior and the full characterization of the slab’s flexural performance up to failure.

### Mesh generation and solver settings

The model was discretized using a structured meshing approach to achieve an optimal balance between computational accuracy and efficiency. To capture the high stress gradients and complex mechanical interactions expected in specific regions, mesh refinement was strategically implemented in critical zones, including the vicinities of the CFRP termination points, the composite interface between the precast bottom slab and the cast-in-situ concrete layer, and the mid-span region where the maximum bending moment occurs. This localized refinement ensures adequate resolution of stress concentrations and potential failure mechanisms in these areas.

In the solver configuration, the geometric nonlinearity option was activated to account for large deformation effects that may arise during the loading process, particularly in the post-yield and post-peak stages. An automatic incremental step algorithm was employed to facilitate stable and efficient solution progression. The initial increment size, minimum increment size, and maximum increment size were appropriately specified to balance computational cost with convergence reliability, allowing the solution to adaptively adjust step sizes in response to the nonlinearity and convergence behavior throughout the analysis.

## Validation of the finite element model

### Validation scheme

The flexural behavior of CFRP-strengthened reinforced concrete beams shares fundamental similarities with that of CFRP-strengthened composite slabs without overextending reinforcing bars, particularly in terms of load transfer mechanisms, failure modes, and the contribution of externally bonded CFRP to flexural capacity. Both are flexural members; a one-way slab behaves essentially as a wide beam. Hence, validation against beam tests provides a rigorous and conservative benchmark for the modeling framework applied to slabs. Given the absence of directly applicable experimental data for the specific slab configuration under investigation, existing experimental results from CFRP-strengthened RC beam tests were selected as a benchmark for validating the finite element modeling approach.

The validation process involved a comprehensive comparison between the finite element simulation results and the corresponding experimental measurements. Key performance indicators, including the load-displacement curves, yield load, ultimate load-bearing capacity, and maximum mid-span deflection, were extracted from both sources and systematically evaluated. The finite element model was considered valid and reliable for subsequent parametric studies if the relative errors between the simulated and experimental values for these key indicators remained within 10%, a commonly accepted tolerance in computational structural analysis. This validation approach ensures that the established modeling techniques, constitutive relationships, and interaction definitions adequately capture the essential mechanical behavior of CFRP-strengthened concrete flexural members.

### Benchmark

To validate the finite element modeling approach adopted in this study, the CFRP-strengthened reinforced concrete beams reported in Reference [16] were selected as the benchmark. The strengthening method employed in the reference experimental program involved externally bonding CFRP sheets to the tension face (bottom surface) of the beams. The test beams had a rectangular cross-section measuring 100 mm in width and 200 mm in height, with a total length of 2500 mm and a clear span of 2400 mm. Longitudinal tension reinforcement consisted of HRB400 steel bars with a diameter of 14 mm, and the concrete cover thickness was maintained at 25 mm. Both the stirrups and the longitudinal compression reinforcement were also fabricated using HRB400 steel bars.

The material properties reported from the experimental tests were as follows: the average cubic compressive strength of concrete was 38.77 MPa; the average yield strength and average ultimate strength of the HRB400 steel bars were 488 MPa and 581 MPa, respectively; the CFRP sheets exhibited a tensile strength of 3700 MPa, a tensile elastic modulus of 2.3 × 10⁵ MPa, and an elongation at break of 1.67%. These experimentally determined material parameters were directly adopted as input data for the corresponding finite element model to ensure consistency between the simulation and the benchmark tests.

### Validation of the CFRP-strengthened reinforced concrete beam model

Based on the aforementioned constitutive models, interaction definitions, and boundary conditions, and in accordance with the loading scheme described in Reference [22], a three-dimensional solid nonlinear finite element model was established using ABAQUS software. To quantitatively verify the accuracy and reliability of the numerical model, the experimental values of yield load, ultimate load, and maximum mid-span deflection obtained from the four test beams with identical parameters in Reference [22] were systematically compared with the corresponding finite element simulation results.

The load-displacement curves derived from both experimental measurements and numerical simulations comprehensively capture the entire flexural response of the beams from initial loading through to failure, clearly delineating the key behavioral stages, including the elastic phase, concrete cracking, reinforcement yielding, and ultimate failure. Characteristic points on these curves—particularly the yield load, ultimate load-bearing capacity, and maximum mid-span deflection—serve as critical indicators for evaluating structural performance and validating the accuracy of the finite element model.

**Fig 3:**
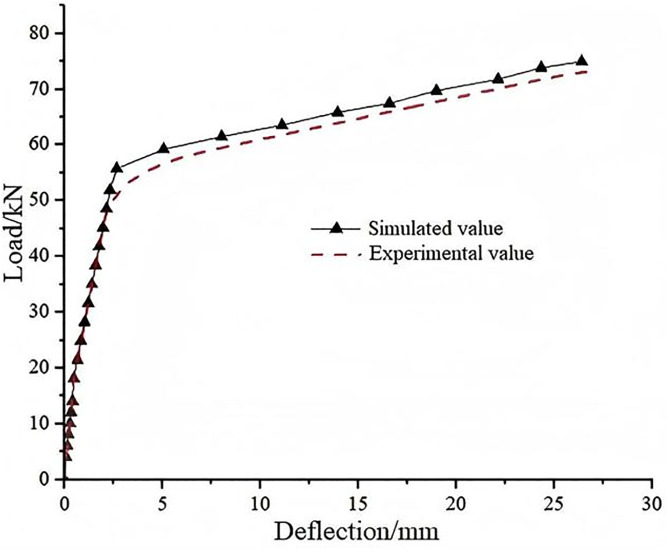
Comparison of simulated and experimental load-deflection curves.

Fig 3 presents a comparative illustration of the load-deflection curves obtained from the experiments and the finite element simulation. It should be noted that specimens C3, C4, C5, and C6 in Reference [22] were fabricated and tested under identical conditions; therefore, the experimental curve shown in Fig 3 represents the average of the test results from these four beams, thereby minimizing the influence of individual specimen variability and providing a more reliable benchmark for validation. The determination of the yield point should satisfy two fundamental principles. First, the yield point should reflect the critical condition at which a local region or the entire member enters the plastic state. Second, the yield point should represent the upper serviceability limit of the member, ensuring both structural safety and applicability. Based on these principles, the yield loads of all specimens were determined using the farthest-point method, as referenced in [29].

The flexural performance parameters of the CFRP-strengthened reinforced concrete beams, determined from the load-deflection curves in Fig 3, are summarized in Table 1.

**Table 1:** Finite element simulation values and experimental results.

Yield Load/kN		Ultimate Load/kN		Maximum Deflection/mm		Experiment-to-Simulation Ratio		
Experimental	Simulated	Experimental	Simulated	Experimental	Simulated	Yield Load	Ultimate Load	Max. Deflection
55.35	53.6	74.95	73.21	25.45	26.9	1.03	1.02	0.95

As can be observed from Fig 3 and Table 2, the load-deflection curves obtained from the finite element simulations demonstrate good agreement with the experimentally measured curves, with the errors in all flexural performance parameters remaining within 5.0%. This level of accuracy confirms the reliability of the modeling approach and falls well within the acceptable range for engineering validation purposes. The results demonstrate that the aforementioned finite element model, incorporating the selected constitutive relationships, interaction definitions, and boundary conditions, is capable of adequately simulating the flexural behavior of both CFRP-strengthened RC beams and the composite slabs under investigation. Consequently, the established modeling framework is deemed suitable for conducting subsequent parametric analyses and for investigating the underlying strengthening mechanisms of CFRP-strengthened composite slabs without overextending reinforcing bars.

**Table 2:** Finite element simulation results.

No.	Thickness /mm	Layer number	Bonding length /mm	Yield Load /kN	Yield displacement /mm
A0-0–0	0	0	0	72.8	6.2
B0-0–0	0	0	0	63.7	7.8
B1-1–500	0.111	1	500	76.5	5.9
B2-1–500	0.167	1	500	81.4	5.4
B1-2–500	0.222	2	500	82.5	5.6
B1-3–500	0.333	3	500	83.2.	5.5
B1-4–500	0.444	4	500	83.9	5.4
B1-2–400	0.222	2	400	79.8	5.7
B1-2–600	0.222	2	600	83.6	5.5
B1-2–700	0.222	2	700	84.5	5.4

Note: All specimens in the B-series feature a joint as depicted in Fig 2, where the longitudinal reinforcement of the precast bottom slab is discontinuous at the joint. The yield displacement refers to the deflection corresponding to the yield load.

## Parametric finite element analysis

### Parametric analysis scheme

To thoroughly investigate the influence of CFRP strengthening configurations on the mechanical performance of composite slabs without overextending reinforcing bars, a systematic parametric analysis was conducted based on the validated finite element model. This analysis examined the effects of key parameters—including the number of CFRP layers, CFRP thickness, and bonding length—on the strengthening effectiveness and overall flexural behavior of the slabs.

In the numerical simulations, the width of each CFRP strip was set to 150 mm, with a clear spacing of 300 mm between adjacent strips, corresponding to a center-to-center spacing of 375 mm. For illustrative purposes, a bonding length of 500 mm represents the total bonded length distributed equally on both sides of the joint, i.e., 250 mm on each side. The analysis was terminated when the slab’s deflection increased to a level at which the ABAQUS solver either failed to achieve convergence or exhibited a sharp decline in convergence rate, indicating numerical instability associated with extensive damage or failure. It should be noted that the load corresponding to the maximum deflection at the point of termination does not necessarily represent the true ultimate load-bearing capacity of the specimen; therefore, the ultimate load-bearing capacity of the specimens was not analyzed in this study. The complete set of simulation results, including yield loads and corresponding displacements for each configuration, is summarized in Table 2.

### Influence of strengthening thickness

**Fig 4:**
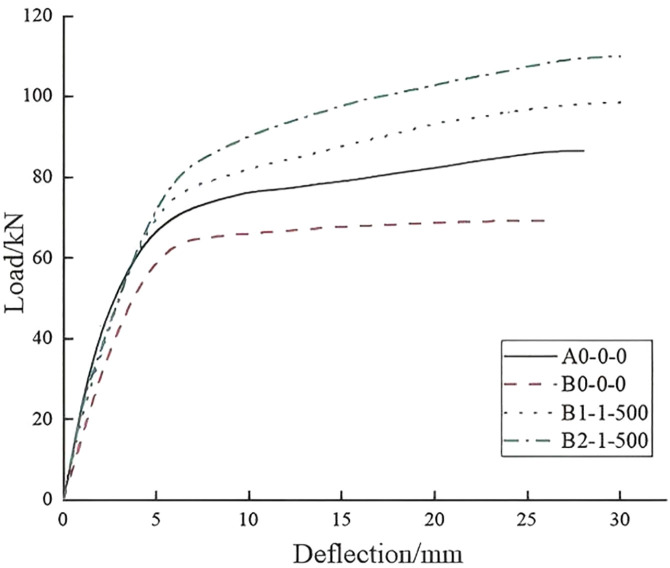
Influence of strengthening thickness on the load-deflection curves.

Fig 4 presents the mid-span deflection curves of specimens A0-0–0, B0-0–0, B1-1–500, and B2-1–500 under vertical loading.

For specimen A0-0–0, which represents a composite slab without joints, the load-deflection curve demonstrates a linear ascending trend during the initial loading stage, exhibiting characteristic elastic behavior. Upon reaching a load of 72.8 kN, a distinct inflection point appears on the curve, corresponding to a yield displacement of 6.2 mm. Thereafter, the slab transitions into the elasto-plastic stage, during which the rate of deflection growth accelerates progressively.

Specimen B0-0–0 is a composite slab without overextending reinforcing bars but incorporating a joint at the mid-span. Its load-bearing process follows a pattern similar to that of A0-0–0, also displaying an initial linear response. When the applied load attains 63.7 kN, an inflection point emerges on the curve, with a corresponding yield displacement of 7.8 mm. In comparison to specimen A0-0–0, the yield load of B0-0–0 decreases by approximately 12.5%, while the yield displacement increases by about 25.8%. This comparative analysis indicates that the presence of a joint significantly reduces both the stiffness and load-bearing capacity of the composite slab, highlighting the critical role of continuity in flexural performance.

Specimens B1-1–500 and B2-1–500 are strengthened with CFRP thicknesses of 0.111 mm and 0.167 mm, respectively. Both specimens exhibit a linear load-deflection relationship during the initial loading phase. Their yield loads are 76.5 kN and 81.4 kN, corresponding to yield displacements of 5.9 mm and 5.4 mm, respectively. Compared to the jointless reference specimen A0-0–0, the yield load of B1-1–500 increases by approximately 5.1%, while its yield displacement decreases by about 4.8%. For B2-1–500, the yield load increases by approximately 14.6%, and the yield displacement decreases by about 12.9%.

The finite element simulation results demonstrate that CFRP strengthening effectively restores the stress transfer path across the joint region through a bridging mechanism. This restoration enables the precast bottom slab—which, in the absence of strengthening, does not participate in load-bearing—to resume its structural function, thereby significantly enhancing both the stiffness and load-bearing capacity of the composite slab system. In the unstrengthened slab without overextending reinforcing bars, the discontinuity of both concrete and longitudinal reinforcement at the joint prevents effective transfer of tensile stress across this critical region. Consequently, the tension zone relies primarily on the steel trusses embedded within the cast-in-situ layer to carry tensile forces, resulting in a reduced internal lever arm for the cross-section. Under such conditions, the precast bottom slab merely serves as permanent formwork and contributes negligibly to the overall load-carrying mechanism.

Following CFRP strengthening, the externally bonded fiber fabric establishes a continuous force-transfer pathway across the joint area, indirectly connecting the discontinuous longitudinal reinforcement on either side of the joint and reinstating the load-bearing contribution of the precast bottom slab. As the thickness of the CFRP reinforcement increases, the yield load of the composite slab exhibits consistent improvement, accompanied by a corresponding reduction in yield displacement. This trend indicates that increasing the strengthening thickness positively influences the structural performance during the elastic stage, enhancing stiffness and delaying the onset of inelastic deformation.

### Influence of strengthening layer number

**Fig 5:**
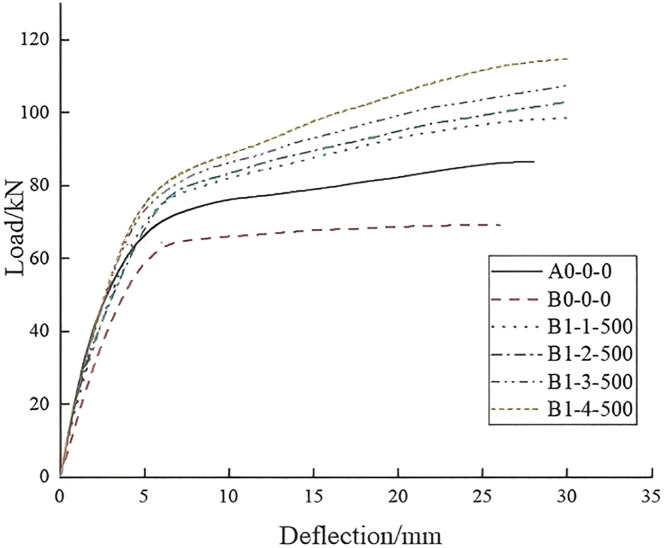
Influence of the number of strengthening layers on the load-deflection curves.

Fig 5 presents the mid-span load-deflection curves for specimens A0-0–0, B0-0–0, B1-1–500, B1-2–500, B1-3–500, and B1-4–500 under monotonically increasing vertical loading. These curves illustrate the influence of varying the number of CFRP strengthening layers on the flexural response of the composite slabs, while maintaining a constant single-layer thickness of 0.111 mm and a bonding length of 500 mm.

As shown in Fig 5, the load-deflection curves for specimens B1-1–500, B1-2–500, B1-3–500, and B1-4–500—strengthened with one, two, three, and four layers of CFRP, respectively—exhibit a consistent bilinear response characterized by an initial linear elastic stage followed by a distinct nonlinear stage. This pattern confirms that all strengthened specimens undergo a pronounced elastic phase followed by an elasto-plastic phase during the loading process, regardless of the number of CFRP layers applied.

Quantitative comparisons with the jointless benchmark specimen A0-0–0 reveal the following improvements in flexural performance. For specimen B1-1–500 (single-layer strengthening), the yield load reaches 76.5 kN with a corresponding yield displacement of 5.9 mm, representing a 5.1% increase in yield load and a 4.8% reduction in yield displacement relative to the benchmark. For B1-2–500 (two-layer strengthening), the yield load increases to 82.5 kN with a yield displacement of 5.6 mm, corresponding to improvements of 13.3% and 9.7%, respectively. For B1-3–500 (three-layer strengthening), the yield load attains 83.2 kN with a yield displacement of 5.5 mm, representing increases of 14.3% in yield load and 11.3% in yield displacement reduction. For B1-4–500 (four-layer strengthening), the yield load reaches 83.9 kN with a yield displacement of 5.4 mm, corresponding to enhancements of 15.2% and 12.9%, respectively.

Analysis of these simulation results reveals an important trend: while increasing the number of CFRP layers consistently improves both the yield load and stiffness (as evidenced by reduced yield displacements) of the strengthened composite slabs, the incremental benefit diminishes as the layer count increases. The transition from one to two layers yields a substantial improvement of approximately 8.2 percentage points in yield load, whereas the subsequent increase from two to three layers contributes only an additional 1.0 percentage point, and from three to four layers merely 0.9 percentage points. This diminishing returns pattern indicates that beyond a certain threshold, further increases in the number of CFRP layers produce limited additional enhancement in structural yield capacity. This observation confirms the existence of a saturation effect, whereby the effectiveness of additional CFRP layers in improving load-bearing capacity becomes progressively less pronounced once a sufficient level of strengthening has been achieved.

### Influence of bonding length

**Fig 6:**
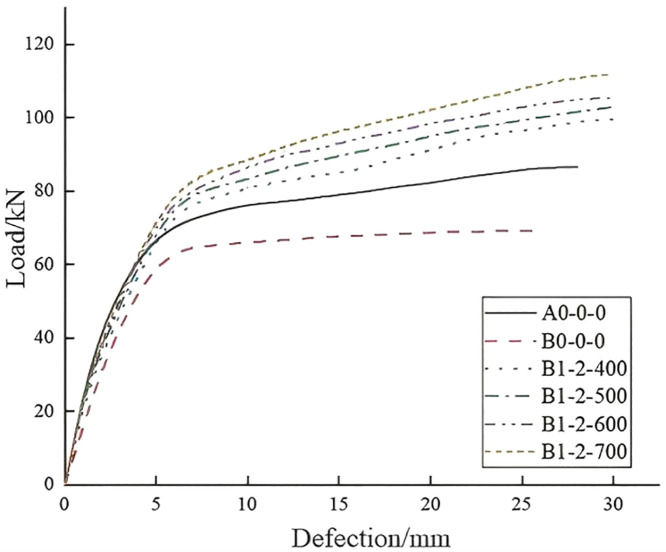
Influence of bonding length on the load-deflection curves.

Fig 6 presents the mid-span load-deflection curves for specimens A0-0–0, B0-0–0, B1-2–400, B1-2–500, B1-2–600, and B1-2–700 under monotonically increasing vertical loading. These curves illustrate the influence of varying CFRP bonding length on the flexural response of the composite slabs, while maintaining a constant CFRP thickness of 0.222 mm (two layers) and a strip width of 150 mm. The bonding lengths investigated range from 400 mm to 700 mm, corresponding to 200 mm to 350 mm on each side of the joint, enabling a systematic evaluation of how this key parameter affects the load-deflection behavior, yield characteristics, and overall structural performance of the CFRP-strengthened composite slabs without overextending reinforcing bars.

For specimens B1-2–400, B1-2–500, B1-2–600, and B1-2–700, the thickness of a single CFRP layer is 0.111 mm, the number of strengthening layers is consistently two, and the bonding lengths are 400 mm, 500 mm, 600 mm, and 700 mm, respectively. As illustrated in Fig 6, the load-deflection curves for all these CFRP-strengthened composite slabs exhibit two distinct stages: a pronounced linear elastic stage followed by a well-defined nonlinear stage, confirming that the specimens undergo a clear elastic phase followed by elasto-plastic deformation during the loading process.

Quantitative comparisons with the jointless benchmark specimen A0-0–0 reveal the following improvements in flexural performance. For specimen B1-2–400 (bonding length of 400 mm), the yield load reaches 79.8 kN with a corresponding yield displacement of 5.7 mm, representing a 9.6% increase in yield load and an 8.1% reduction in yield displacement relative to the benchmark. For B1-2–500 (500 mm bonding length), the yield load increases to 82.5 kN with a yield displacement of 5.6 mm, corresponding to improvements of 13.3% and 9.7%, respectively. For B1-2–600 (600 mm bonding length), the yield load attains 83.6 kN with a yield displacement of 5.5 mm, representing increases of 14.8% in yield load and 11.3% in yield displacement reduction. For B1-2–700 (700 mm bonding length), the yield load reaches 84.5 kN with a yield displacement of 5.4 mm, corresponding to enhancements of 16.1% and 12.9%, respectively.

Analysis of these simulation results reveals a critical insight regarding the influence of bonding length on flexural performance. While all strengthened specimens demonstrate improved load-bearing capacity and reduced yield displacements compared to the unstrengthened jointed specimen, the incremental benefit of increasing bonding length exhibits a diminishing trend. The improvement in yield load progresses from 9.6% to 13.3% as bonding length increases from 400 mm to 500 mm, but further extensions to 600 mm and 700 mm yield progressively smaller marginal gains of only 1.5 and 1.3 percentage points, respectively. This pattern indicates that once the bonding length exceeds a certain threshold—approximately 500 mm in this investigation—further increases in bonding length contribute only marginally to enhancing the load-bearing capacity.

The simulation results thus demonstrate a clear saturation effect of bonding length on yield capacity. When the bonding length reaches a value sufficient to fully develop the stress transfer between the CFRP and the concrete substrate—corresponding to the bond stress transfer length of the reinforcement in the concrete—additional bonding length beyond this critical value provides limited further improvement in structural performance. This finding has important practical implications, suggesting that an optimal bonding length exists beyond which further extension yields diminishing returns in terms of enhanced load-bearing capacity.

## Mechanical behavior and design method for CFRP-strengthened composite slabs without overextending reinforcing bars

### Mechanical behavior

The connection in composite slabs with overextending reinforcing bars fundamentally constitutes a “wet connection” between the reinforcing bars embedded in the precast slab and the subsequently placed cast-in-situ concrete, thereby forming a rigid monolithic joint. This connection detail effectively enables the transfer of tensile stress carried by the longitudinal reinforcement in the precast bottom slab across the joint region. Under vertical loading, the internal lever arm of the normal section is defined as the distance from the centroid of the compression zone to the centroid of the longitudinal reinforcement in the precast bottom slab, which governs the flexural capacity of the section.

**Fig 7:**
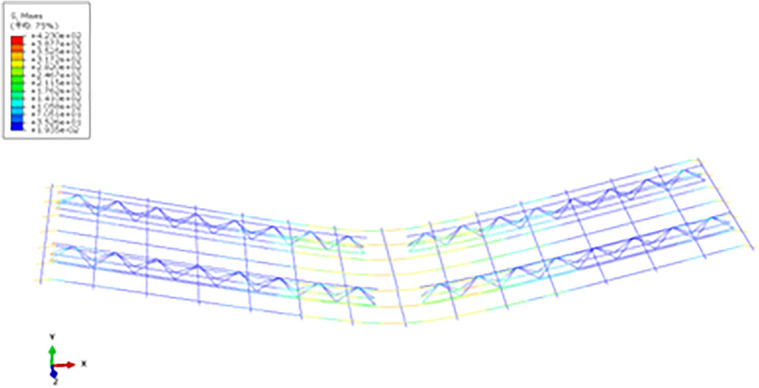
Reinforcement stress distribution in specimen B0-0-0.

In contrast, for composite slabs without overextending reinforcing bars, the presence of joints introduces discontinuities in both the longitudinal reinforcement and the concrete of the precast bottom slab at these locations. This discontinuity precludes the effective transfer of tensile stress across the joint region, rendering the precast bottom slab unable to participate in the overall load-resisting mechanism. As a result, the tension zone relies primarily on the steel trusses within the cast-in-situ layer to carry tensile forces, leading to a reduced internal lever arm and, consequently, a lower load-bearing capacity compared to conventional slabs with overextending reinforcing bars. A comparative illustration of the stress distributions in these two configurations is presented in Fig 7.

**Fig 8:**
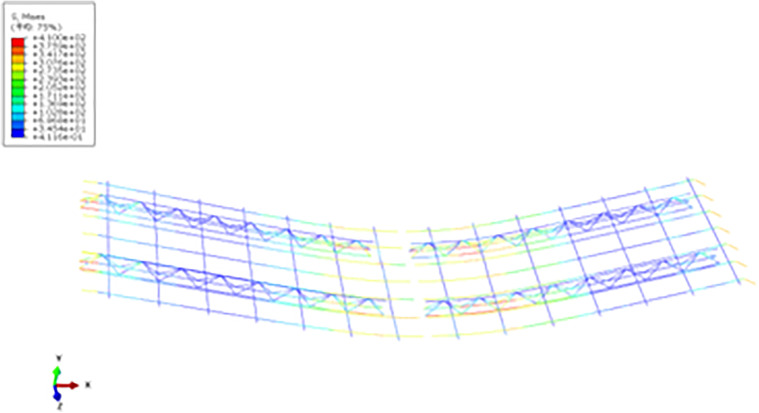
Reinforcement stress distribution of specimen B1-2-500.

The fundamental strengthening mechanism of CFRP in composite slabs without overextending reinforcing bars lies in its bridging effect across the discontinuous reinforcement at the joint. Although the longitudinal reinforcement within the precast bottom slab is interrupted at the joint location, the tensile stresses that develop in this critical region are effectively carried by the externally bonded CFRP and transferred to the concrete of the precast slab. Subsequently, through the bond action at the concrete-steel interface, these stresses are further transmitted to the longitudinal reinforcement embedded within the precast bottom slab. This load transfer mechanism enables the CFRP to indirectly connect the discontinuous reinforcement on both sides of the joint, thereby restoring the continuity of force transmission across the joint region. The resulting stress distribution, illustrating this indirect force transfer path, is schematically represented in Fig 8.

The efficiency of force transfer in the CFRP strengthening system is critically dependent on the adequacy of its bonding length. If the provided bonding length falls short of the bond stress transfer length required for the reinforcement embedded in the concrete, the interfacial bond stresses between the CFRP and the concrete substrate may attain their peak prematurely. This premature stress concentration can precipitate either debonding failure at the CFRP-concrete interface or tensile cracking of the concrete cover, thereby preventing the full utilization of the CFRP’s strengthening potential and limiting the effectiveness of the retrofit.

Conversely, once the bonding length satisfies or exceeds the requisite bond stress transfer length, tensile stresses can be effectively transmitted across the joint region and into the longitudinal reinforcement of the precast bottom slab through the sequential load transfer mechanism described previously. However, as demonstrated by the simulation results presented in Fig 6, extending the CFRP bonding length beyond this necessary threshold does not yield commensurate improvements in the ultimate load-bearing capacity of the slab. The marginal gains in yield load diminish progressively with further increases in bonding length, confirming the existence of an effective saturation point.

Therefore, in design practice, the specified bonding length for CFRP strengthening should establish a lower bound that reliably exceeds the bond stress transfer length of the longitudinal reinforcement within the concrete. This minimum requirement ensures that the full tensile capacity of the CFRP can be mobilized before interfacial debonding or other premature failure modes occur. Simultaneously, the selected bonding length must comply with relevant detailing requirements stipulated in applicable design codes and standards, thereby balancing structural performance with practical construction considerations.

### Design method

The design of CFRP-strengthened composite slabs without overextending reinforcing bars must satisfy two fundamental requirements: equal-strength force transfer and effective bond force transfer. These principles ensure that the externally bonded CFRP reinforcement can adequately substitute for the interrupted longitudinal reinforcement at the joint region and reliably transmit tensile forces across the discontinuity [30–32].

According to the equal-strength principle, the tensile capacity provided by the CFRP reinforcement should not be less than the tensile capacity of the longitudinal reinforcement in the precast bottom slab. This requirement guarantees that the CFRP can fully replicate the interrupted force-transfer function of the reinforcement at the joint, thereby restoring the continuity of the tension force path and enabling the precast bottom slab to resume its load-bearing role within the composite section. The equal-strength condition is expressed mathematically by Equation (12):


$$\begin{array}{c} n_{\text{f}}b_{\text{f}}t_{\text{f}}f_{\text{f}}/s_{\text{f}}\geq f_{\text{y}}A_{\text{s}}/s \end{array}$$ (12)

where, $n_{\text{f}}$ is the number of CFRP layers; $b_{\text{f}}$ is the width of a CFRP strip; $t_{\text{f}}$ is the thickness of a single CFRP layer; $f_{\text{f}}$ is the design tensile strength of the CFRP; $s_{\text{f}}$ is the center-to-center spacing of CFRP strips; $f_{\text{y}}$ is the design strength of the longitudinal reinforcement in the precast bottom slab; $A_{\text{s}}$ is the cross-sectional area of a single longitudinal reinforcing bar in the precast bottom slab; s is the spacing of the longitudinal reinforcement in the precast bottom slab.

Based on the effective force transfer principle, the bonding length of the CFRP reinforcement must simultaneously satisfy two essential requirements: first, the bond anchorage length between the CFRP and the concrete substrate of the precast bottom slab must be sufficient to develop the full tensile capacity of the composite material; and second, the basic anchorage length required for the longitudinal reinforcement embedded within the precast bottom slab concrete must also be satisfied to ensure adequate stress transfer from the CFRP through the concrete to the interrupted reinforcement.

These dual requirements arise from the indirect nature of the force transfer mechanism, wherein tensile stresses are transmitted from the CFRP to the concrete via interfacial bond, and subsequently from the concrete to the longitudinal reinforcement through steel-concrete bond action. Consequently, the design bonding length must be sufficient to accommodate both bond-dependent load transfer processes. The governing bonding length should therefore be determined as the larger of these two required anchorage lengths, as expressed in Equation (13):


$$\begin{array}{c} l\geq 2⬝max\left(l_{\text{f}},l_{\text{a}}\right) \end{array}$$ (13)

where, $l_{\text{f}}$ is the designed anchorage length between CFRP and the precast bottom slab concrete, as specified in the the Chinese national standard Code for Design of Strengthening Concrete Structures (GB 50367−2013); $l_{\text{a}}$ is the designed anchorage length of the reinforcement in the precast bottom slab concrete, as specified in the Chinese national standard General Code for Concrete Structures (GB 55008−2021).

The aforementioned mechanical behavior analysis reveals that CFRP serves primarily as an indirect bridging element in composite slabs without overextending reinforcing bars, functioning essentially as a localized strengthening intervention at the joint region. Consequently, an effective strengthening threshold exists: once the tensile capacity provided by the CFRP reinforcement and its associated bonding length are sufficient to achieve adequate stress transfer across the joint discontinuity, further increases in CFRP thickness, number of layers, or bonding length contribute only marginally to enhancing the overall flexural capacity of the slab. This saturation effect, consistently observed in the parametric analysis results, underscores the importance of identifying optimal strengthening configurations rather than pursuing indiscriminate increases in reinforcement quantity.

In light of this finding, after completing the local strengthening design in accordance with the equal-strength and effective bond transfer principles outlined above, the calculation of the overall flexural capacity and stiffness of the CFRP-strengthened composite slab without overextending reinforcing bars can be performed using the same methodology applicable to traditional composite slabs with overextending reinforcing bars (i.e., “wet connection” slabs). This equivalence arises because, once the joint region is adequately strengthened, the composite slab effectively behaves as a continuous flexural member, with the precast bottom slab fully engaged in the load-resisting mechanism. Therefore, conventional section analysis methods, based on strain compatibility and equilibrium considerations, become applicable for predicting the flexural performance of the strengthened system.

## Conclusions and discussions

By establishing a refined three-dimensional finite element model using the ABAQUS platform, this study systematically investigated the flexural performance of CFRP-strengthened composite slabs without overextending reinforcing bars. The influence of key parameters—including CFRP thickness, number of layers, and bonding length—on the yield behavior and deformation characteristics of the slabs was thoroughly analyzed, and the underlying strengthening mechanism was elucidated. Based on the results obtained, the following main conclusions are drawn:

(1) CFRP strengthening effectively restores the stress transfer path at the joint region of composite slabs without overextending reinforcing bars through a bridging mechanism, thereby significantly enhancing both the yield load and flexural stiffness of the structural system. In comparison with unstrengthened jointed specimens, the yield load of CFRP-strengthened slabs can be increased by up to approximately 16.1%, accompanied by a corresponding reduction in yield displacement of about 12.9%, indicating substantial improvements in both load-carrying capacity and elastic stiffness.
(2) Increasing the CFRP thickness and the number of strengthening layers enhances the load-bearing capacity of the composite slabs within a certain range. However, as the number of layers continues to increase, the rate of improvement in yield capacity progressively diminishes, exhibiting a pronounced saturation effect. For the single-layer thickness of 0.111 mm adopted in this study, strengthening with two layers restores most of the structural performance achievable through CFRP reinforcement; further increasing the layer count to three or four yields only marginal additional benefits, suggesting that an optimal layer configuration exists beyond which further reinforcement becomes inefficient.
(3) The bonding length of CFRP reinforcement exerts a significant influence on force transfer efficiency up to a critical threshold. Once the bonding length surpasses the bond stress transfer length required for the longitudinal reinforcement embedded in the concrete, further extension of the bonding length does not produce significant enhancements in structural load-bearing capacity. This finding confirms the existence of an effective critical bonding length, beyond which additional bond length contributes little to improving flexural performance.
(4) Drawing upon the parametric analysis results, a comprehensive CFRP strengthening design methodology centered on two fundamental principles—equal-strength force transfer and effective bond force transfer—is proposed. Specific formulas for determining the required CFRP cross-sectional area and the minimum bonding length are established. In consideration of the saturation effects observed for CFRP thickness, number of layers, and bonding length, and taking into account economic efficiency, it is recommended that design and construction satisfy only the minimum values determined by the equal-strength and effective force transfer principles, in conjunction with relevant code-specified detailing requirements. This approach ensures adequate structural performance while avoiding unnecessary material consumption and construction costs.

The finite element analysis presented in this study provides valuable insights into the flexural behavior of CFRP-strengthened composite slabs without overextending reinforcing bars. However, several limitations and areas for improvement should be acknowledged to guide future research and enhance the robustness and applicability of the findings.

Firstly, while the manuscript mentions mesh refinement in critical regions such as the CFRP termination points and the slab joint, it does not include a formal mesh sensitivity or convergence study. Such an analysis is essential to verify that the simulation results are independent of the mesh size and that the chosen discretization adequately captures stress gradients without introducing numerical artifacts. Future studies should systematically vary mesh density and report convergence behavior to strengthen the reliability of the numerical predictions.

Secondly, the CFRP–concrete interface is modeled using cohesive elements with specified parameters for stiffness and fracture energy. However, the calibration procedure and justification for these parameters are not clearly described. The values adopted should be supported by experimental data or referenced from established literature. Without such justification, the accuracy of the simulated debonding behavior and interfacial stress transfer remains uncertain. Future work should include experimental validation of interface properties or parametric studies to assess the sensitivity of the model to these parameters.

Thirdly, the current study focuses primarily on global response indicators such as load-deflection curves and yield loads. While these are important for assessing structural performance, a more detailed discussion of failure mechanisms would enhance the depth of the analysis. For instance, contour plots of concrete damage, CFRP stress distribution, and interfacial debonding progression could provide valuable insight into the sequence and nature of failure. Such information is critical for understanding how and why strengthening becomes less effective beyond certain thresholds, and for validating the numerical model against experimental observations.

Lastly, although the manuscript identifies a saturation effect in the effectiveness of increasing CFRP layers and bonding length, the mechanical explanation for this behavior could be expanded. A more in-depth interpretation—considering factors such as strain redistribution, stress concentration at the CFRP ends, and the limited force transfer capacity of the interface—would strengthen the discussion. Additionally, the interaction between CFRP layers and the embedded steel reinforcement, as well as the role of concrete cracking in limiting stress transfer, deserves further exploration.

In summary, while this study provides a useful foundation for understanding the flexural performance of CFRP-strengthened composite slabs without overextending reinforcing bars, future research should address the limitations outlined above. Specifically, experimental validation, mesh sensitivity analysis, detailed failure characterization, and deeper mechanical interpretation are recommended to advance the knowledge base and support the practical application of this innovative structural form.

## Supporting information

S1 File ABAQUS CAE model file. (ZIP)

S2 File ABAQUS ODB file. (ZIP)
