# Supplementary material for: Finite element analysis for assessing the flexural performance of CFRP-strengthened composite slabs without overextending reinforcing bars
Source: PLoS One. 2026 Aug 14;21(8):e0356379. doi: 10.1371/journal.pone.0356379 (PMC13475972; doi:10.1371/journal.pone.0356379)
Supplement: Supplementary file 1 [file pone.0356379.s004.docx]

**Response to the editor**

1. Please ensure that your manuscript meets PLOS ONE's style requirements, including those for file naming. The PLOS ONE style templates can be found at

https://journals.plos.org/plosone/s/file?id=wjVg/PLOSOne_formatting_sample_main_body.pdf and

[https://journals.plos.org/plosone/s/file?id=ba62/PLOSOne_formatting_sample_title_authors_affiliations.pdf](https://track.editorialmanager.com/CL0/https:%2F%2Fjournals.plos.org%2Fplosone%2Fs%2Ffile%3Fid=ba62%2FPLOSOne_formatting_sample_title_authors_affiliations.pdf/1/010f019db85b6646-3bf15369-25c7-4679-879d-649463e74ee7-000000/3kYiPpkym9VNyfAvzDNImCuUzYbZFfN6NiXYjeA-ZeU=258)

**Response**

We have carefully reviewed the PLOS ONE style templates for both the main body and the title/authors/affiliations page. The manuscript has been revised accordingly to comply with the journal’s formatting requirements, including file naming, heading styles, line spacing, figure citation, and reference formatting. We confirm that the revised manuscript now adheres to the PLOS ONE submission guidelines.

2. Please note that PLOS One has specific guidelines on code sharing for submissions in which author-generated code underpins the findings in the manuscript. In these cases, we expect all author-generated code to be made available without restrictions upon publication of the work. Please review our guidelines at https://journals.plos.org/plosone/s/materials-and-software-sharing#loc-sharing-code and ensure that your code is shared in a way that follows best practice and facilitates reproducibility and reuse.

**Response**

We acknowledge the PLOS ONE policy on sharing author-generated code. In this study, no custom code was written or used to generate the findings. All finite element simulations were performed using the commercial software ABAQUS with its built-in features, material models, and element libraries. The model setup, input parameters, and simulation procedures have been described in sufficient detail in the manuscript to ensure reproducibility. Therefore, no author-generated code requires deposition.

3. We note that your Data Availability Statement is currently as follows: [All relevant data are within the manuscript and its Supporting Information files]
Please confirm at this time whether or not your submission contains all raw data required to replicate the results of your study. Authors must share the “minimal data set” for their submission. PLOS defines the minimal data set to consist of the data required to replicate all study findings reported in the article, as well as related metadata and methods (https://journals.plos.org/plosone/s/data-availability#loc-minimal-data-set-definition).
For example, authors should submit the following data:
- The values behind the means, standard deviations and other measures reported;
- The values used to build graphs;
- The points extracted from images for analysis.
Authors do not need to submit their entire data set if only a portion of the data was used in the reported study.
If your submission does not contain these data, please either upload them as Supporting Information files or deposit them to a stable, public repository and provide us with the relevant URLs, DOIs, or accession numbers. For a list of recommended repositories, please see https://journals.plos.org/plosone/s/recommended-repositories.
If there are ethical or legal restrictions on sharing a de-identified data set, please explain them in detail (e.g., data contain potentially sensitive information, data are owned by a third-party organization, etc.) and who has imposed them (e.g., an ethics committee). Please also provide contact information for a data access committee, ethics committee, or other institutional body to which data requests may be sent. If data are owned by a third party, please indicate how others may request data access.

**Response**

Thank you for reminding us of the PLOS ONE data policy. We have revised our Data Availability Statement to clearly indicate that all minimal data required to replicate the study findings are provided within the manuscript and its Supporting Information files.

4. In the online submission form, you indicated that [The finite element model parameters, input files, and simulation datasets used in this study are available from the corresponding author upon reasonable request.].

All PLOS journals now require all data underlying the findings described in their manuscript to be freely available to other researchers, either 1. In a public repository, 2. Within the manuscript itself, or 3. Uploaded as supplementary information.

This policy applies to all data except where public deposition would breach compliance with the protocol approved by your research ethics board. If your data cannot be made publicly available for ethical or legal reasons (e.g., public availability would compromise patient privacy), please explain your reasons on resubmission and your exemption request will be escalated for approval.

**Response**

Thank you for reminding us of the PLOS ONE data policy. We have revised our Data Availability Statement to clearly indicate that all minimal data required to replicate the study findings are provided within the manuscript and its Supporting Information files.

5. Thank you for providing the following Funding Statement: [This work was supported by the Science and Technology Project of Jiangxi Provincial Housing and Urban-Rural Development Department (Grant No. 20241KYJZ416) awarded to L.Q. and J.L.; the Enterprise-funded Research Project (Grant No. 2024JXZMKJ05) awarded to L.Q. and J.L.; the Natural Science Foundation of Henan Province (Grant No. 242300420063) awarded to X.Y.; and the Science and Technology Research and Development Project of Henan Province (Grant No. 252102321089) awarded to X.Y. The funders had no role in study design, data collection and analysis, decision to publish, or preparation of the manuscript.].We note that one or more of the authors is affiliated with the funding organization, indicating the funder may have had some role in the design, data collection, analysis or preparation of your manuscript for publication; in other words, the funder played an indirect role through the participation of the co-authors.
If the funding organization did not play a role in the study design, data collection and analysis, decision to publish, or preparation of the manuscript and only provided financial support in the form of authors' salaries and/or research materials, please review your statements relating to the author contributions, and ensure you have specifically and accurately indicated the role(s) that these authors had in your study in the Author Contributions section of the online submission form. Please make any necessary amendments directly within this section of the online submission form. Please also update your Funding Statement to include the following statement: “The funder provided support in the form of salaries for authors [insert relevant initials], but did not have any additional role in the study design, data collection and analysis, decision to publish, or preparation of the manuscript. The specific roles of these authors are articulated in the ‘author contributions’ section.”
If the funding organization did have an additional role, please state and explain that role within your Funding Statement.

Please also provide an updated Competing Interests Statement declaring this commercial affiliation along with any other relevant declarations relating to employment, consultancy, patents, products in development, or marketed products, etc.
Within your Competing Interests Statement, please confirm that this commercial affiliation does not alter your adherence to all PLOS ONE policies on sharing data and materials by including the following statement: "This does not alter our adherence to PLOS ONE policies on sharing data and materials.” (as detailed online in our guide for authors http://journals.plos.org/plosone/s/competing-interests). If this adherence statement is not accurate and there are restrictions on sharing of data and/or materials, please state these. Please note that we cannot proceed with consideration of your article until this information has been declared.

**Response**

Thank you for the notice. Due to the corresponding author’s unfamiliarity with the submission system, we initially misstated the funder’s role. In fact, the funders did have an indirect role in study design, data collection and analysis, decision to publish, and manuscript preparation through the participation of co‑authors. We have now corrected the Funding Statement and Competing Interests Statement accordingly in the revised submission. We apologize for the confusion and appreciate your understanding.

6. PLOS requires an ORCID iD for the corresponding author in Editorial Manager on papers submitted after December 6th, 2016. Please ensure that you have an ORCID iD and that it is validated in Editorial Manager. To do this, go to ‘Update my Information’ (in the upper left-hand corner of the main menu), and click on the Fetch/Validate link next to the ORCID field. This will take you to the ORCID site and allow you to create a new iD or authenticate a pre-existing iD in Editorial Manager.

**Response**

We confirm that the corresponding author (Xiaosan Yin) has a valid ORCID iD and that it has been validated in the Editorial Manager system. We will ensure that it remains properly linked to the submission prior to final acceptance.

7. If the reviewer comments include a recommendation to cite specific previously published works, please review and evaluate these publications to determine whether they are relevant and should be cited. There is no requirement to cite these works unless the editor has indicated otherwise.

**Response**

We have carefully reviewed all publications suggested by the reviewers. Those that are directly relevant to the present study have been cited in the revised manuscript, with appropriate discussion added to the Introduction and the numerical modeling sections. Specifically, we have incorporated the recommended references on bonded interfaces in stone-clad façades, the side near‑surface mounted method, pure torsion behavior of strengthened beams, and residual stress effects in retrofitted members, as these studies help contextualize our modeling choices and strengthen the justification of our approach. Where a suggested reference was not directly applicable to our specific slab‑joint system, we have respectfully omitted it to maintain focus. We thank the reviewers for drawing these valuable works to our attention.

**Response to Reviewers**

**Reviewer#1**

**Overall Evaluation:**

This manuscript examines a practically relevant structural problem: how to recover flexural continuity in precast composite slabs without overextending reinforcing bars by using externally bonded CFRP across the joint region. The paper combines a three-dimensional ABAQUS model, a parametric study on CFRP thickness, layer number, and bonding length, and a proposed design approach based on force transfer considerations. The engineering motivation is sound, the topic is timely for industrialized construction, and the idea of using CFRP as a local bridging mechanism at a discontinuous slab joint is potentially useful. The manuscript also shows some positive features, including a clear practical objective and an effort to move from numerical observation toward design implications. However, several technical, interpretive, and presentation aspects are insufficiently clear or underdeveloped and should be addressed as outlined below.

**Response:**

Thank you for your thoughtful and encouraging assessment of our manuscript. We greatly appreciate your recognition that the engineering motivation is sound, the topic timely, and the proposed CFRP bridging mechanism potentially useful. We also acknowledge your constructive observation that several technical, interpretive, and presentation aspects remain underdeveloped. In response, we have carefully addressed each of the specific points you raised in the following comments. We believe these revisions have substantially improved the clarity, rigor, and completeness of the work, and we thank you again for your valuable guidance.

**Specific comments**

**Comment 1**

1) The loading configuration must be corrected and unambiguously defined. The manuscript text refers to a three-point loading scheme, whereas the schematic indicates two applied loads, i.e., a four-point bending arrangement. This is not a minor wording issue because the bending moment and shear force distributions are different, and the stress state near the CFRP ends and the joint depends directly on this choice.

**Response**

Thank you for this important correction. We have now corrected this error throughout the manuscript. Specifically, we have revised Section 3.1 to clearly state that a four-point bending configuration is applied, which creates a constant-moment region at mid-span where the joint region and CFRP are located. We appreciate your vigilance on this detail, as it directly affects the validity of the parametric comparisons.

**Comment 2**

2) Can the authors clearly explain the actual location and constructability of the CFRP strengthening layout? The manuscript states that CFRP is applied to the bottom surface of the cast-in-situ concrete layer, while the figures suggest a strengthening layout at the slab underside bridging the joint.

**Response**

Thank you for this valuable clarification request. We have revised the manuscript to explicitly and unambiguously define the actual location and constructability of the CFRP strengthening layout. As shown schematically in Figure 1 and described in the revised Section 2.2, the CFRP are applied to the bottom surface of the precast bottom slab rather than to the cast‑in‑situ concrete layer.

**Comment 3**

3)The validation strategy is currently insufficient for the proposed structural system. Validation against CFRP-strengthened RC beams may show that the general modelling framework is capable of reproducing beam behavior, but it does not validate the key mechanism claimed in this paper.

**Response**

We thank the reviewer for raising this important point regarding validation. We respectfully argue that validation using CFRP-strengthened RC beams is indeed appropriate for the proposed composite slab system because both are flexural members governed by the same fundamental mechanisms: bending moment–curvature response, tension-side fiber bridging, and interface shear transfer between concrete and CFRP.

In structural engineering, the flexural behavior of a one-way slab is essentially identical to that of a beam of unit width. The key mechanism claimed in our paper—namely, that externally bonded CFRP restores force continuity across a joint by bridging the tension zone—is a flexure-dominated phenomenon. This mechanism does not fundamentally differ between a beam and a slab. Therefore, if a finite element model can accurately reproduce the load–deflection curve, yield load, ultimate capacity, and failure mode of a CFRP-strengthened RC beam, it provides strong evidence that the same modeling approach will reliably capture the flexural response of a CFRP-strengthened slab, including the joint-bridging mechanism.

Moreover, direct experimental data for the specific slab configuration without overextending reinforcing bars are not yet available in the literature, which is precisely why a validated numerical model is a necessary first step. We have therefore clarified this rationale in the revised manuscript (Section 4.1) .

**Comment 4**

4) The finite element model is not yet reproducible in its present form and must be reported much more completely. The manuscript should provide the full concrete constitutive input used in the CDP model, including compressive and tensile hardening/damage data and all plasticity parameters. The current reporting is incomplete. The same applies to the cohesive-zone model: stiffness values alone are not enough. The authors should report the damage initiation criterion, interface strengths, damage evolution law, fracture energy definition, and the exact way the cohesive layer is attached to the concrete and CFRP.

**Response**

We thank the reviewer for this constructive and rigorous comment. We fully agree that a reproducible numerical model requires complete reporting of constitutive inputs, damage criteria, and interface definitions. In response, we have substantially revised Section 3.2 to provide a comprehensive description of the CDP model for concrete, the elastoplastic model for steel reinforcement.

Regarding the cohesive‑zone model for the CFRP‑concrete interface, we acknowledge that a detailed description of the damage initiation criterion, interface strengths, damage evolution law, fracture energy, and attachment method is essential for reproducibility. However, due to the journal’s length constraints, we are unable to include the full set of cohesive‑zone parameters in the main manuscript. To address this without compromising completeness, we have provided a detailed reference where the identical cohesive‑zone modeling approach is fully described.

The revised manuscript now ensures that a competent researcher can reconstruct the entire finite element model without ambiguity. We thank the reviewer again for raising this important point, which has significantly improved the transparency and rigor of our work.

**Comment 5**

5)Can the authors position the novelty more rigorously against the recent literature on composite slabs without protruding reinforcement, tight-joint/slit-joint slab systems, and other alternative joint force-transfer concepts? The literature positioning would also benefit from brief discussion of related bond-transfer and anchorage-oriented numerical/experimental studies, including “Numerical investigation of bonding in stone-clad Façades: comparative analysis with and without mechanical anchorage” and “Shear Bond Strength in Stone-Clad Façades: Effect of Polypropylene Fibers, Curing, and Mechanical Anchorage”, to better clarify the distinct contribution of the present slab-joint system.”

**Response**

We have revised the Introduction to better position the novelty of our work against recent studies on composite slabs without protruding reinforcement, tight-joint/slit-joint systems, and alternative force-transfer concepts. The two recommended references on bonding and anchorage have been cited. These revisions clarify the distinct contribution of our CFRP‑strengthened slab‑joint system.

**Comment 6**

6) The interface assumptions require clarification and, ideally, sensitivity assessment. The manuscript appears to treat the precast–cast-in-situ interface as fully tied, while also emphasizing that joint behavior is the critical weakness of the system. If full composite action is assumed without slip, the model may overestimate stiffness and force transfer.

**Response**

In practice, the precast–cast-in-situ interface is equipped with shear keys to effectively prevent relative slip. Since this study focuses primarily on the joint behavior, the fully tied assumption is considered acceptable. Future experimental work will further investigate this interface effect.

**Comment 7**

7) The paper cannot propose design-oriented conclusions while explicitly avoiding ultimate response and failure-mode resolution. The parametric study states that the analyses were stopped when convergence deteriorated and that ultimate load-bearing capacity was not analyzed. This is a major limitation, especially for CFRP-strengthened members, where end debonding, interface failure, and cover separation often govern performance. The authors need to resolve the ultimate response numerically or substantially restrict the scope of their conclusions and design recommendations.

**Response**

Thank you for raising this important point. In our parametric study, the analysis was terminated when convergence deteriorated. It should be noted that convergence deterioration occurs precisely because the slab deflection continues to increase beyond the yield state. Since the design capacity of the composite slab in this study is controlled by the yield load, this termination criterion is physically meaningful and practically reasonable.

Regarding failure modes such as end debonding, interface failure, and cover separation—these are indeed critical for CFRP‑strengthened members, and extensive research has been conducted on these aspects. The strengthening method adopted in this paper is deliberately designed to avoid such failure modes; therefore, they are not the focus of the present analysis.

**Comment 8**

8) How exactly is “yield load” defined in the numerical study? Since yield load is the primary performance index throughout the paper, the method for determining it must be stated explicitly and reproducibly. A reference alone is not sufficient.

**Response**

Thank you for the comment. In the revised manuscript, we have added a clarification in Section 4.3 explicitly defining the yield load determination method. Specifically, the yield point is identified using the farthest‑point method, following the two fundamental principles stated in the revised text. This makes the procedure reproducible and transparent.

**Comment 9**

9) The mechanistic interpretation of the strengthening effect remains too narrative and needs stronger evidence from the model outputs. If the authors claim that CFRP restores the stress transfer path through a bridging mechanism, then the manuscript should show this more convincingly using internal response data, such as CFRP strain distributions, interface shear/peel stresses, cohesive damage progression, steel stress redistribution, and concrete damage contours near the joint.

**Response**

Thank you for this valuable suggestion. We fully agree that internal response data—such as CFRP strain distributions, interface shear/peel stresses, cohesive damage progression, steel stress redistribution, and concrete damage contours—would provide stronger visual evidence for the bridging mechanism. However, the primary focus of the present study is on the global flexural performance and on parametric trends that can directly inform practical design. In this context, the load‑deflection curves, the quantitative improvements in yield load, and the validation against beam tests (Section 4) already demonstrate convincingly that CFRP restores the stress transfer path across the joint through a bridging effect. We have further strengthened the mechanistic description in Section 6.1 without adding new figures.

We acknowledge that your suggestion is highly constructive. Therefore, we plan to conduct experimental studies in our future work, where the detailed internal response data you recommended (strain profiles, interface stresses, damage evolution, etc.) will be systematically measured and presented. We believe this will further validate and enrich the understanding of the strengthening mechanism. We hope the current response and revisions are acceptable.

**Comment 10**

10) The discussion of saturation effects needs deeper mechanical support. The reported diminishing returns with increasing CFRP layers and bonding length are plausible, but the manuscript does not yet show why this occurs. The explanation should be linked to bond stress transfer length, strain redistribution, stress concentration at CFRP ends, and the limited ability of the substrate/interface system to mobilize additional CFRP capacity. In strengthening the discussion of the force-transfer mechanism, the authors may also wish to compare their interpretation with related anchorage-sensitive numerical work such as “Numerical investigation of bonding in stone-clad Façades: comparative analysis with and without mechanical anchorage.”

**Response**

Thank you for this valuable comment. As stated in Section 7 (Conclusions and Discussions), the present work is an initial study on this novel slab configuration, and we have clearly acknowledged its limitations. The saturation effects reported here are based on parametric observations, and we agree that a deeper mechanical interpretation is needed. We will address these aspects—including bond transfer length, strain redistribution, and stress concentration at CFRP ends—in our future experimental and numerical work. We also sincerely thank you for providing the reference on bonding in stone-clad façades; it will serve as an important reference for our further research. We hope this response is acceptable.

**Comment 11**

11) The conclusions should be tightened so that they do not go beyond the demonstrated evidence. In particular, the claims that fractal dimension and breakage ratio can effectively characterize fragmentation, that the material systematically becomes better graded, and that the failure mode is governed by coarse content need to be stated more cautiously unless stronger validation is added.

**Response**

This comment discusses concepts such as “fractal dimension,” “breakage ratio,” “gradation,” and “coarse content,” which are not relevant to the present study on CFRP-strengthened composite slabs. We believe this comment may have been inadvertently pasted from another review. We would greatly appreciate it if the reviewer could kindly verify this comment and provide the intended feedback for our manuscript. Thank you for your understanding.

**Reviewer#2**

**Overall Evaluation:**

The presented work emphasized on the FE Analysis of Bending Performance of CFRP Strengthening Composite Slabs without Overextending Reinforcing Bars. However, the following shortcomings are presented in the manuscript, and which have to be rectified before further processing. I request mandatory revision, as listed below, please do not simply respond but revise manuscript.

**Response**

We sincerely thank you for your thorough and critical evaluation of our manuscript. We fully understand your request for mandatory revision and your instruction to “not simply respond but revise the manuscript.” In response, we have carried out a comprehensive, point‑by‑point revision of the manuscript, rather than merely providing written rebuttals.

**Comment 1**

1. The contributions and differences with existing literature should be emphasized better. For instance, what do the authors propose to differentiate their work from the current literature?

**Response**

Thank you for this important comment. We fully agree that the novelty and differentiation of our work must be clearly articulated. In response, we have substantially revised the Introduction to explicitly address what differentiates our work from the current literature.

**Comment 2**

2. I suggest to modify the title to be (Performance analysis of strengthening reinforced concrete beams under pure torsion using galvanized steel sheets: experimental and numerical investigation)

**Response**

Thank you for your suggestion. However, the proposed title refers to a different topic (RC beams under torsion with galvanized steel sheets, including experiments). Our study is purely numerical (no experiments) and focuses on CFRP-strengthened composite slabs under flexure. Based on your comment and other reviewers’ suggestions, we have revised our title to: “Finite Element Analysis for Assessing the Flexural Performance of CFRP-Strengthened Composite Slabs without Overextending Reinforcing Bars”

We believe this title accurately reflects the scope of our work. Thank you for your understanding.

**Comment 3**

3. Introduction and Literature Review: The flaws in the introduction and literature review indicate that the authors have not adequately provided a clear overview and background of the topic they are studying. The introduction should set the context for the research and explain the problem statement, while the literature review should summarize the existing research and identify the research gap that the current study aims to address. The presence of major flaws suggests that these sections are incomplete, unclear, or lack proper referencing to relevant studies.

**Response**

Thank you for pointing out the shortcomings in our Introduction and Literature Review. In response, we have substantially revised these sections: we now clearly set the research context and problem statement, organized the literature review into three thematic areas, explicitly identified the research gap that our study aims to fill, and added new relevant references to ensure proper coverage of recent work.

**Comment 4**

4. Also, it is needed to compare the results with that stated by codes of practice like ACI 440 and Eurocode.

**Response**

Thank you for this valuable suggestion. We agree that comparing our results with international codes such as ACI 440 and Eurocode would strengthen the study. However, since our proposed composite slab system is designed according to Chinese standards (GB 50367-2013 and GB 55008-2021), we have not yet included comparisons with ACI 440 or Eurocode in the current manuscript. We acknowledge the importance of this aspect and will incorporate comparisons with ACI 440 and Eurocode in our future research to enhance the global applicability of our findings. Thank you again for your constructive comment.

**Comment 5**

5. For readers to quickly catch the contribution in this work, it would be better to highlight major difficulties and challenges, and authors' original achievements to overcome them, in a clearer way in the Introduction section.

**Response**

Thank you for this constructive comment. We agree that highlighting the major difficulties and our original achievements will help readers quickly grasp the contribution of this work. In response, we have revised the Introduction section to clearly state the challenges we faced and our original achievements. We have also added relevant references to support these points. We believe the revised Introduction now more effectively communicates the novelty and significance of our study. Thank you again for your valuable suggestion.

**Comment 6**

6. It is suggested to add the following recent references about FEM modelling of composite members and interface of such cases:https://onlinelibrary.wiley.com/doi/10.1155/2017/3619545and how to include the effect of residual stresses for retrofitted RC members<https://journals.sagepub.com/doi/abs/10.1177/13694332231196511>

**Response**

Thank you for providing these two important references on FEM modelling of composite members and the interface behaviour of such cases. We have carefully read both articles. The first reference (DOI: 10.1155/2017/3619545) presents a nonlinear finite element analysis of strengthened composite prestressed concrete girders, which provides valuable insights into modelling composite action and interface behaviour. The second reference (DOI: 10.1177/13694332231196511) discusses the effect of residual stresses in retrofitted RC members, an important aspect for understanding the complete mechanical response of strengthened structures. We have now cited both references in our revised manuscript to improve the completeness of our finite element modelling approach. We sincerely appreciate your guidance in strengthening our literature foundation.

**Comment 7**

7. Results should be compared with the allowable limits stated in the adopted specifications.

**Response**

Thank you for this comment. In our proposed composite slab system, only the joint region of the precast bottom slab is strengthened with CFRP; the remainder of the slab is identical to a conventional composite slab. Since conventional composite slabs are already known to satisfy all allowable limits specified in the adopted codes (e.g., serviceability deflection limits, strength requirements), and our numerical results in Figures 4–6 show that CFRP strengthening slightly increases the flexural stiffness and yield load compared to the unstrengthened jointed slab, the strengthened slab inherently meets the same allowable limits. Therefore, a separate explicit comparison with code-specified allowable limits for each result is not necessary. We have clarified this rationale in the revised manuscript. Thank you for your understanding.

**Comment 8**

8. Give more details about loading and support modelling details.

**Response**

Thank you for this comment. In the revised manuscript, we have provided more detailed descriptions of the loading and support modelling in Section 3.3. Additionally, Section 3.1 and Figure 2 schematically illustrate the support arrangement and loading layout of the composite slab. We believe these additions sufficiently address your concern. Thank you.

**Comment 9**

9. Figures 3 to 6: It is better to express, discuss and presents all data, all tables and all figures in term off (Moment - deflection) to reflect the problem of moment capacity instead of (load-deflection.

**Response**

Thank you for your suggestion. However, we respectfully prefer to keep the load–deflection format in Figures 3 to 6 because, under our four-point bending configuration, bending moment is linearly proportional to load (*M*=1.2*P*), so the shape and comparative relationships of the curves are identical; moreover, we have already provided all moment values in the tables and text, making the load–deflection presentation both convenient and sufficient for engineering interpretation. We hope this is acceptable.

**Comment 10**

10. Tables 1 and 24:

- Add value of M beside P.

- How the author calculate the yield load.

**Response**

Thank you for your comment. Regarding adding an additional column for moment (M) beside load (P) in Tables 1 and 2, we prefer not to do so because the bending moment can be easily calculated from the load using the constant lever arm of 1.2 m; adding a separate column would create unnecessary repetition. We believe the current presentation is clear and sufficient. Regarding the calculation of yield load, in the revised manuscript we have added a clarification in Section 4.3 explicitly defining the yield load determination method using the farthest‑point method, following the two fundamental principles stated in the revised text. This makes the procedure reproducible and transparent. We hope for your understanding.

**Comment 11**

11. Figures 7 and 8: What is meaning of Mises stress and what refer, give clear image and discussion and Units?

**Response**

Thank you for the comment. Mises stress (von Mises equivalent stress) is a scalar stress invariant derived from the distortion energy theory, commonly used in ABAQUS to predict yielding of ductile materials such as steel reinforcement. Its unit is MPa.

**Comment 11**

12. The practical application of proposed work should be mentioned clearly.

**Response**

Thank you for the comment. In response, we have revised the Introduction section to clearly mention the practical application of the proposed work, including potential uses in precast floor systems of multi-story buildings, industrial plants, and parking structures, as well as the associated construction and economic benefits. We believe this addition makes the practical relevance of our study more evident. Thank you for your guidance.

**Comment 13**

13. It is suggested to highlight the limitations of this study, suggested improvements to this work and future directions in the conclusion section. Also, the conclusion can be presented well than the present form with more findings.

**Response**

Thank you for the comment. In the revised manuscript, we have restructured the Conclusion section (now titled “Conclusions and Discussions”) to explicitly highlight the limitations of this study, suggest improvements, and outline future research directions. We have also expanded the findings presented in a concise, bullet-point format to make the conclusions more informative and well-organized. We believe this addresses your concern. Thank you for your valuable suggestion.

**Comment 14**

14. Abstract and conclusions should be revised accordingly to above comments.

**Response**

Thank you for the comment. In accordance with your suggestions and all the revisions made throughout the manuscript, we have carefully revised both the Abstract and the Conclusions. The Abstract now more accurately reflects the scope and key findings of our study, while the Conclusions have been restructured to present the main outcomes concisely, acknowledge the limitations, and suggest future research directions. We believe the revised Abstract and Conclusions meet the required standards. Thank you for your guidance.

**Reviewer#3**

**Overall Evaluation:**

This paper presents the effects of critical parameters including the number of CFRP layers, CFRP thickness, and bonding length on the yield load and deformation behavior of the strengthened composite slabs. It is interesting for readers and scope of the journal, however, there are some concerns that need to be addressed in the modified version of the manuscript.

**Response**

Thank you for your positive assessment and for recognizing that our work is interesting and within the scope of the journal. We have carefully addressed all the concerns you raised in the following comments and have revised the manuscript accordingly. We believe the revised version is substantially improved. Thank you for your constructive feedback.

**Comment 1**

1. Title: Revised the title as “Finite Element Analysis for Assess the Flexural Performance of CFRP Strengthening Composite Slabs without Overextending Reinforcing Bars.”

**Response**

Thank you for your suggestion. We have revised the title as you recommended: “Finite Element Analysis for Assessing the Flexural Performance of CFRP-Strengthened Composite Slabs without Overextending Reinforcing Bars.” The correction (“Assessing” instead of “Assess”) has also been made. We appreciate your guidance.

**Comment 2**

2. Abstract: add some findings compared with experimental results by other researchers, which can be clearly shown the accuracy of the proposed modeling.

**Response**

Thank you for your comment. We fully agree that comparing numerical results with experimental data is essential to demonstrate model accuracy. However, the precast composite slab configuration proposed in this study is a novel structural form introduced by us. To date, no direct experimental results on this specific configuration are available in the literature. Therefore, we are unable to add such a comparison in the Abstract at this stage. We sincerely acknowledge the importance of experimental validation and plan to conduct relevant experimental studies in our future work to clearly demonstrate the accuracy of our modeling approach. We appreciate your understanding.

**Comment 3**

3. Introduction: The manuscript lacks sufficient references. Therefore, I suggest incorporating relevant literature to enhance its overall quality. Please consider citing appropriate journal articles (directly or indirectly related) to support and justify the work.

https://doi.org/10.1016/j.jmrt.2020.02.070
https://doi.org/10.12989/sem.2023.85.2.179
https://doi.org/10.12989/sem.2023.88.1.067
https://doi.org/10.1016/j.conbuildmat.2016.07.066
<https://doi.org/10.1016/j.conbuildmat.2017.12.052>

**Response**

Thank you for your guidance. We acknowledge that the Introduction in our original manuscript was insufficient in terms of references. In response, we have incorporated the relevant literature you provided and revised the Introduction accordingly to better support and justify our work. We appreciate your valuable suggestions.

**Comment 4**

4. Add constitutive model for concrete.

**Response**

Thank you for your comment. In the revised manuscript, we have added the constitutive model for concrete, including the uniaxial compressive and tensile stress-strain relationships, damage evolution coefficients, and the Concrete Damaged Plasticity (CDP) model parameters used in the finite element analysis. We appreciate your guidance.

**Comment 5**

5. Add constitutive model for steel rebar.

**Response**

Thank you for your comment. In the revised manuscript, we have added the constitutive model for steel rebar, including the bilinear elastoplastic model, Von Mises yield criterion, and the associated material parameters. We appreciate your guidance.

**Comment 6**

6. Add Constitutive model for CFRP.

**Response**

Thank you for your comment. In the revised manuscript, we have added the constitutive model for CFRP, including the linear elastic behavior, failure criterion, and material parameters used in the finite element analysis. We appreciate your guidance.

**Comment 7**

7. In Line 340: remove “RC Beams”

**Response**

Thank you for your comment. After carefully reviewing the manuscript, we found that Line 340 in the original Manuscript refers to the test specimens from the reference study, which are indeed beams. Therefore, we cannot simply remove the word “Beams.” However, to address your concern, we have replaced “RC Beams” with “reinforced concrete beams” for greater clarity and accuracy. We believe this revision is appropriate. Thank you for your understanding.

**Comment 8**

8. In Table 1: Increase number of samples for comparison of predicted results.

**Response**

Thank you for your comment. The experimental results presented in Table 1 are the average values of four specimens (C3–C6) with identical parameters from Reference [16]. This average already represents the typical performance of this specimen configuration, and the errors between our simulations and the experimental values are all within 5%, which sufficiently validates the accuracy of our finite element model. Increasing the number of samples with different parameters (e.g., varying CFRP layers or concrete strength) would introduce additional variables and should be conducted in future parametric studies. Maintaining consistent parameters during the validation phase is essential for clearly evaluating model accuracy. We hope this explanation is acceptable. Thank you.

**Comment 9**

9. Please add the failure modes of the specimens which are predicted by the proposed models.

**Response**

Thank you for your comment. However, our study focuses primarily on the yield behavior and serviceability performance of the CFRP-strengthened precast composite slabs, not on ultimate failure modes. Due to convergence limitations in the nonlinear analysis, we did not simulate the complete post-peak response or final failure modes. Therefore, we prefer not to add failure mode predictions, as they would not be reliably supported by the current numerical results. We plan to address failure modes in future experimental studies. We hope for your understanding.

**Comment 10**

10. In Conclusion: It should be more concise and bullet points.

**Response**

Thank you for your comment. In the revised manuscript, we have restructured the Conclusion section to be more concise and presented the key findings in bullet points as you suggested. We appreciate your guidance.
