# Supplementary material for: Finite element analysis for assessing the flexural performance of CFRP-strengthened composite slabs without overextending reinforcing bars
Source: PLoS One. 2026 Aug 14;21(8):e0356379. doi: 10.1371/journal.pone.0356379 (PMC13475972; doi:10.1371/journal.pone.0356379)
Supplement: Supplementary file 2 [file pone.0356379.s001.zip › S1 File. ABAQUS CAE model file/README – Supporting Information File.docx]

**README – Supporting Information File**

This file is a finite element model created with **ABAQUS (version 2024)**. It is provided to facilitate the reproduction of the finite element analysis results reported in the accompanying manuscript.

Please be aware that this file **can only be opened and viewed using the ABAQUS software** (including the ABAQUS/CAE or ABAQUS/Viewer modules). Users without access to ABAQUS will not be able to open or inspect its contents.

Should you require more detailed information about the model setup, boundary conditions, or material properties, or if you encounter any issues accessing the file, please do not hesitate to contact the corresponding author at: **yinxiaosan_3@126.com**.

Thank you for your interest in our work.
