# Supplementary material for: Finite element analysis for assessing the flexural performance of CFRP-strengthened composite slabs without overextending reinforcing bars
Source: PLoS One. 2026 Aug 14;21(8):e0356379. doi: 10.1371/journal.pone.0356379 (PMC13475972; doi:10.1371/journal.pone.0356379)
Supplement: Peer review history

# Peer review: Decision Letter 0

**DOI:** 10.1371/journal.pone.0356379.r001

**Authors:** Antonio Riveiro Rodríguez

22 Apr 2026

Dear Dr. Yin,

Please submit your revised manuscript by Jun 06 2026 11:59PM. If you will need more time than this to complete your revisions, please reply to this message or contact the journal office at plosone@plos.org. When you're ready to submit your revision, log on to https://www.editorialmanager.com/pone/ and select the 'Submissions Needing Revision' folder to locate your manuscript file.

- A letter that responds to each point raised by the academic editor and reviewer(s). You should upload this letter as a separate file labeled 'Response to Reviewers'.
- A marked-up copy of your manuscript that highlights changes made to the original version. You should upload this as a separate file labeled 'Revised Manuscript with Track Changes'.
- An unmarked version of your revised paper without tracked changes. You should upload this as a separate file labeled 'Manuscript'.

If you would like to make changes to your financial disclosure, please include your updated statement in your cover letter. Guidelines for resubmitting your figure files are available below the reviewer comments at the end of this letter.

If applicable, we recommend that you deposit your laboratory protocols in protocols.io to enhance the reproducibility of your results. Protocols.io assigns your protocol its own identifier (DOI) so that it can be cited independently in the future. For instructions see: https://journals.plos.org/plosone/s/submission-guidelines#loc-laboratory-protocols. Additionally, PLOS ONE offers an option for publishing peer-reviewed Lab Protocol articles, which describe protocols hosted on protocols.io. Read more information on sharing protocols at https://plos.org/protocols?utm_medium=editorial-email&utm_source=authorletters&utm_campaign=protocols.

As the corresponding author, your ORCID iD is verified in the submission system and will appear in the published article. PLOS supports the use of ORCID, and we encourage all coauthors to register for an ORCID iD and use it as well. Please encourage your coauthors to verify their ORCID iD within the submission system before final acceptance, as unverified ORCID iDs will not appear in the published article. *Only*  the individual author can complete the verification step; PLOS staff *cannot*  verify ORCID iDs on behalf of authors.

We look forward to receiving your revised manuscript.

Kind regards,

Antonio Riveiro Rodríguez, PhD

Academic Editor

PLOS One

Journal Requirements:

When submitting your revision, we need you to address these additional requirements.

1. Please ensure that your manuscript meets PLOS ONE's style requirements, including those for file naming. The PLOS ONE style templates can be found at

https://journals.plos.org/plosone/s/file?id=wjVg/PLOSOne_formatting_sample_main_body.pdf and

https://journals.plos.org/plosone/s/file?id=ba62/PLOSOne_formatting_sample_title_authors_affiliations.pdf

2. Please note that PLOS One has specific guidelines on code sharing for submissions in which author-generated code underpins the findings in the manuscript. In these cases, we expect all author-generated code to be made available without restrictions upon publication of the work. Please review our guidelines at https://journals.plos.org/plosone/s/materials-and-software-sharing#loc-sharing-code and ensure that your code is shared in a way that follows best practice and facilitates reproducibility and reuse.

3. We note that your Data Availability Statement is currently as follows: [All relevant data are within the manuscript and its Supporting Information files]

Please confirm at this time whether or not your submission contains all raw data required to replicate the results of your study. Authors must share the “minimal data set” for their submission. PLOS defines the minimal data set to consist of the data required to replicate all study findings reported in the article, as well as related metadata and methods (https://journals.plos.org/plosone/s/data-availability#loc-minimal-data-set-definition).

For example, authors should submit the following data:

- The values behind the means, standard deviations and other measures reported;

- The values used to build graphs;

- The points extracted from images for analysis.

Authors do not need to submit their entire data set if only a portion of the data was used in the reported study.

If your submission does not contain these data, please either upload them as Supporting Information files or deposit them to a stable, public repository and provide us with the relevant URLs, DOIs, or accession numbers. For a list of recommended repositories, please see https://journals.plos.org/plosone/s/recommended-repositories.

If there are ethical or legal restrictions on sharing a de-identified data set, please explain them in detail (e.g., data contain potentially sensitive information, data are owned by a third-party organization, etc.) and who has imposed them (e.g., an ethics committee). Please also provide contact information for a data access committee, ethics committee, or other institutional body to which data requests may be sent. If data are owned by a third party, please indicate how others may request data access.

4. In the online submission form, you indicated that [The finite element model parameters, input files, and simulation datasets used in this study are available from the corresponding author upon reasonable request.].

All PLOS journals now require all data underlying the findings described in their manuscript to be freely available to other researchers, either 1. In a public repository, 2. Within the manuscript itself, or 3. Uploaded as supplementary information.

This policy applies to all data except where public deposition would breach compliance with the protocol approved by your research ethics board. If your data cannot be made publicly available for ethical or legal reasons (e.g., public availability would compromise patient privacy), please explain your reasons on resubmission and your exemption request will be escalated for approval.

5. Thank you for providing the following Funding Statement: [This work was supported by the Science and Technology Project of Jiangxi Provincial Housing and Urban-Rural Development Department (Grant No. 20241KYJZ416) awarded to L.Q. and J.L.; the Enterprise-funded Research Project (Grant No. 2024JXZMKJ05) awarded to L.Q. and J.L.; the Natural Science Foundation of Henan Province (Grant No. 242300420063) awarded to X.Y.; and the Science and Technology Research and Development Project of Henan Province (Grant No. 252102321089) awarded to X.Y. The funders had no role in study design, data collection and analysis, decision to publish, or preparation of the manuscript.].

We note that one or more of the authors is affiliated with the funding organization, indicating the funder may have had some role in the design, data collection, analysis or preparation of your manuscript for publication; in other words, the funder played an indirect role through the participation of the co-authors.

If the funding organization did not play a role in the study design, data collection and analysis, decision to publish, or preparation of the manuscript and only provided financial support in the form of authors' salaries and/or research materials, please review your statements relating to the author contributions, and ensure you have specifically and accurately indicated the role(s) that these authors had in your study in the Author Contributions section of the online submission form. Please make any necessary amendments directly within this section of the online submission form. Please also update your Funding Statement to include the following statement: “The funder provided support in the form of salaries for authors [insert relevant initials], but did not have any additional role in the study design, data collection and analysis, decision to publish, or preparation of the manuscript. The specific roles of these authors are articulated in the ‘author contributions’ section.”

If the funding organization did have an additional role, please state and explain that role within your Funding Statement.

Please also provide an updated Competing Interests Statement declaring this commercial affiliation along with any other relevant declarations relating to employment, consultancy, patents, products in development, or marketed products, etc.

Within your Competing Interests Statement, please confirm that this commercial affiliation does not alter your adherence to all PLOS ONE policies on sharing data and materials by including the following statement: "This does not alter our adherence to PLOS ONE policies on sharing data and materials.” (as detailed online in our guide for authors http://journals.plos.org/plosone/s/competing-interests). If this adherence statement is not accurate and there are restrictions on sharing of data and/or materials, please state these. Please note that we cannot proceed with consideration of your article until this information has been declared.

6. PLOS requires an ORCID iD for the corresponding author in Editorial Manager on papers submitted after December 6th, 2016. Please ensure that you have an ORCID iD and that it is validated in Editorial Manager. To do this, go to ‘Update my Information’ (in the upper left-hand corner of the main menu), and click on the Fetch/Validate link next to the ORCID field. This will take you to the ORCID site and allow you to create a new iD or authenticate a pre-existing iD in Editorial Manager.

7. If the reviewer comments include a recommendation to cite specific previously published works, please review and evaluate these publications to determine whether they are relevant and should be cited. There is no requirement to cite these works unless the editor has indicated otherwise.

[Note: HTML markup is below. Please do not edit.]

Reviewers' comments:

Reviewer's Responses to Questions

**Comments to the Author**

1. Is the manuscript technically sound, and do the data support the conclusions?

Reviewer #1: Yes

Reviewer #2: Yes

Reviewer #3: Yes

**********

2. Has the statistical analysis been performed appropriately and rigorously?

Reviewer #1: No

Reviewer #2: Yes

Reviewer #3: Yes

**********

3. Have the authors made all data underlying the findings in their manuscript fully available?

The PLOS Data policy

Reviewer #1: Yes

Reviewer #2: Yes

Reviewer #3: No

**********

4. Is the manuscript presented in an intelligible fashion and written in standard English?

Reviewer #1: No

Reviewer #2: Yes

Reviewer #3: No

**********

Reviewer #1: Overall Evaluation

This manuscript examines a practically relevant structural problem: how to recover flexural continuity in precast composite slabs without overextending reinforcing bars by using externally bonded CFRP across the joint region. The paper combines a three-dimensional ABAQUS model, a parametric study on CFRP thickness, layer number, and bonding length, and a proposed design approach based on force transfer considerations. The engineering motivation is sound, the topic is timely for industrialized construction, and the idea of using CFRP as a local bridging mechanism at a discontinuous slab joint is potentially useful. The manuscript also shows some positive features, including a clear practical objective and an effort to move from numerical observation toward design implications. However, several technical, interpretive, and presentation aspects are insufficiently clear or underdeveloped and should be addressed as outlined below.

Specific comments:

1) The loading configuration must be corrected and unambiguously defined. The manuscript text refers to a three-point loading scheme, whereas the schematic indicates two applied loads, i.e., a four-point bending arrangement. This is not a minor wording issue because the bending moment and shear force distributions are different, and the stress state near the CFRP ends and the joint depends directly on this choice.

2) Can the authors clearly explain the actual location and constructability of the CFRP strengthening layout? The manuscript states that CFRP is applied to the bottom surface of the cast-in-situ concrete layer, while the figures suggest a strengthening layout at the slab underside bridging the joint.

3) The validation strategy is currently insufficient for the proposed structural system. Validation against CFRP-strengthened RC beams may show that the general modelling framework is capable of reproducing beam behavior, but it does not validate the key mechanism claimed in this paper.

4) The finite element model is not yet reproducible in its present form and must be reported much more completely. The manuscript should provide the full concrete constitutive input used in the CDP model, including compressive and tensile hardening/damage data and all plasticity parameters. The current reporting is incomplete. The same applies to the cohesive-zone model: stiffness values alone are not enough. The authors should report the damage initiation criterion, interface strengths, damage evolution law, fracture energy definition, and the exact way the cohesive layer is attached to the concrete and CFRP.

5) Can the authors position the novelty more rigorously against the recent literature on composite slabs without protruding reinforcement, tight-joint/slit-joint slab systems, and other alternative joint force-transfer concepts? The literature positioning would also benefit from brief discussion of related bond-transfer and anchorage-oriented numerical/experimental studies, including “Numerical investigation of bonding in stone-clad Façades: comparative analysis with and without mechanical anchorage” and “Shear Bond Strength in Stone-Clad Façades: Effect of Polypropylene Fibers, Curing, and Mechanical Anchorage”, to better clarify the distinct contribution of the present slab-joint system.”

6) The interface assumptions require clarification and, ideally, sensitivity assessment. The manuscript appears to treat the precast–cast-in-situ interface as fully tied, while also emphasizing that joint behavior is the critical weakness of the system. If full composite action is assumed without slip, the model may overestimate stiffness and force transfer.

7) The paper cannot propose design-oriented conclusions while explicitly avoiding ultimate response and failure-mode resolution. The parametric study states that the analyses were stopped when convergence deteriorated and that ultimate load-bearing capacity was not analyzed. This is a major limitation, especially for CFRP-strengthened members, where end debonding, interface failure, and cover separation often govern performance. The authors need to resolve the ultimate response numerically or substantially restrict the scope of their conclusions and design recommendations.

8) How exactly is “yield load” defined in the numerical study? Since yield load is the primary performance index throughout the paper, the method for determining it must be stated explicitly and reproducibly. A reference alone is not sufficient.

9) The mechanistic interpretation of the strengthening effect remains too narrative and needs stronger evidence from the model outputs. If the authors claim that CFRP restores the stress transfer path through a bridging mechanism, then the manuscript should show this more convincingly using internal response data, such as CFRP strain distributions, interface shear/peel stresses, cohesive damage progression, steel stress redistribution, and concrete damage contours near the joint.

10) The discussion of saturation effects needs deeper mechanical support. The reported diminishing returns with increasing CFRP layers and bonding length are plausible, but the manuscript does not yet show why this occurs. The explanation should be linked to bond stress transfer length, strain redistribution, stress concentration at CFRP ends, and the limited ability of the substrate/interface system to mobilize additional CFRP capacity. In strengthening the discussion of the force-transfer mechanism, the authors may also wish to compare their interpretation with related anchorage-sensitive numerical work such as “Numerical investigation of bonding in stone-clad Façades: comparative analysis with and without mechanical anchorage.”

11) The conclusions should be tightened so that they do not go beyond the demonstrated evidence. In particular, the claims that fractal dimension and breakage ratio can effectively characterize fragmentation, that the material systematically becomes better graded, and that the failure mode is governed by coarse content need to be stated more cautiously unless stronger validation is added.

Reviewer #2: The presented work emphasized on the FE Analysis of Bending Performance of CFRP Strengthening Composite Slabs without Overextending Reinforcing Bars. However, the following shortcomings are presented in the manuscript, and which have to be rectified before further processing. I request mandatory revision, as listed below, please do not simply respond but revise manuscript.

1. The contributions and differences with existing literature should be emphasized better. For instance, what do the authors propose to differentiate their work from the current literature?

2. I suggest to modify the title to be (Performance analysis of strengthening reinforced concrete beams under pure torsion using galvanized steel sheets: experimental and numerical investigation).

3. Introduction and Literature Review: The flaws in the introduction and literature review indicate that the authors have not adequately provided a clear overview and background of the topic they are studying. The introduction should set the context for the research and explain the problem statement, while the literature review should summarize the existing research and identify the research gap that the current study aims to address. The presence of major flaws suggests that these sections are incomplete, unclear, or lack proper referencing to relevant studies.

4. Also, it is needed to compare the results with that stated by codes of practice like ACI 440 and Eurocode.

5. For readers to quickly catch the contribution in this work, it would be better to highlight major difficulties and challenges, and authors' original achievements to overcome them, in a clearer way in the Introduction section.

6. It is suggested to add the following recent references about FEM modelling of composite members and interface of such cases:

https://onlinelibrary.wiley.com/doi/10.1155/2017/3619545

and how to include the effect of residual stresses for retrofitted RC members

https://journals.sagepub.com/doi/abs/10.1177/13694332231196511

7. Results should be compared with the allowable limits stated in the adopted specifications.

8. Give more details about loading and support modelling details.

9. Figures 3 to 6: It is better to express, discuss and presents all data, all tables and all figures in term off (Moment - deflection) to reflect the problem of moment capacity instead of (load-deflection.

10. Tables 1 and 24:

- Add value of M beside P.

- How the author calculate the yield load.

11. Figures 7 and 8: What is meaning of Misses stress and what refer, give clear image and discussion and Units?

12. The practical application of proposed work should be mentioned clearly.

13. It is suggested to highlight the limitations of this study, suggested improvements to this work and future directions in the conclusion section. Also, the conclusion can be presented well than the present form with more findings.

14. Abstract and conclusions should be revised accordingly to above comments.

Reviewer #3: This paper presents the effects of critical parameters including the number of CFRP layers, CFRP thickness, and bonding length on the yield load and deformation behavior of the strengthened composite slabs. It is interesting for readers and scope of the journal, however, there are some concerns that need to be addressed in the modified version of the manuscript.

1. Title: Revised the title as “Finite Element Analysis for Assess the Flexural Performance of CFRP Strengthening Composite Slabs without Overextending Reinforcing Bars.”

2. Abstract: add some findings compared with experimental results by other researchers, which can be clearly shown the accuracy of the proposed modeling.

3. Introduction: The manuscript lacks sufficient references. Therefore, I suggest incorporating relevant literature to enhance its overall quality. Please consider citing appropriate journal articles (directly or indirectly related) to support and justify the work.

https://doi.org/10.1016/j.jmrt.2020.02.070

https://doi.org/10.12989/sem.2023.85.2.179

https://doi.org/10.12989/sem.2023.88.1.067

https://doi.org/10.1016/j.conbuildmat.2016.07.066

https://doi.org/10.1016/j.conbuildmat.2017.12.052

4. Add constitutive model for concrete.

5. Add constitutive model for steel rebar.

6. Add Constitutive model for CFRP.

7. In Line 340: remove “RC Beams”

8. In Table 1: Increase number of samples for comparison of predicted results.

9. Please add the failure modes of the specimens which are predicted by the proposed models.

10. In Conclusion: It should be more concise and bullet points.

**********

what does this mean?). If published, this will include your full peer review and any attached files.

If you choose “no”, your identity will remain anonymous but your review may still be made public.

**Do you want your identity to be public for this peer review?**  For information about this choice, including consent withdrawal, please see our Privacy Policy

Reviewer #1: No

Reviewer #2: **Yes:**  Abbas AbdulMajeed Allawi

Reviewer #3: No

**********

[NOTE: If reviewer comments were submitted as an attachment file, they will be attached to this email and accessible via the submission site. Please log into your account, locate the manuscript record, and check for the action link "View Attachments". If this link does not appear, there are no attachment files.]

To ensure your figures meet our technical requirements, please review our figure guidelines: https://journals.plos.org/plosone/s/figures

You may also use PLOS’s free figure tool, NAAS, to help you prepare publication quality figures: https://journals.plos.org/plosone/s/figures#loc-tools-for-figure-preparation.

NAAS will assess whether your figures meet our technical requirements by comparing each figure against our figure specifications.

# Author comment: Author response to Decision Letter 1

**DOI:** 10.1371/journal.pone.0356379.r002

16 Jul 2026

Response to Reviewers

Reviewer#1

Overall Evaluation:

This manuscript examines a practically relevant structural problem: how to recover flexural continuity in precast composite slabs without overextending reinforcing bars by using externally bonded CFRP across the joint region. The paper combines a three-dimensional ABAQUS model, a parametric study on CFRP thickness, layer number, and bonding length, and a proposed design approach based on force transfer considerations. The engineering motivation is sound, the topic is timely for industrialized construction, and the idea of using CFRP as a local bridging mechanism at a discontinuous slab joint is potentially useful. The manuscript also shows some positive features, including a clear practical objective and an effort to move from numerical observation toward design implications. However, several technical, interpretive, and presentation aspects are insufficiently clear or underdeveloped and should be addressed as outlined below.

Response:

Thank you for your thoughtful and encouraging assessment of our manuscript. We greatly appreciate your recognition that the engineering motivation is sound, the topic timely, and the proposed CFRP bridging mechanism potentially useful. We also acknowledge your constructive observation that several technical, interpretive, and presentation aspects remain underdeveloped. In response, we have carefully addressed each of the specific points you raised in the following comments. We believe these revisions have substantially improved the clarity, rigor, and completeness of the work, and we thank you again for your valuable guidance.

Specific comments

Comment 1

1) The loading configuration must be corrected and unambiguously defined. The manuscript text refers to a three-point loading scheme, whereas the schematic indicates two applied loads, i.e., a four-point bending arrangement. This is not a minor wording issue because the bending moment and shear force distributions are different, and the stress state near the CFRP ends and the joint depends directly on this choice.

Response

Thank you for this important correction. We have now corrected this error throughout the manuscript. Specifically, we have revised Section 3.1 to clearly state that a four-point bending configuration is applied, which creates a constant-moment region at mid-span where the joint region and CFRP are located. We appreciate your vigilance on this detail, as it directly affects the validity of the parametric comparisons.

Comment 2

2) Can the authors clearly explain the actual location and constructability of the CFRP strengthening layout? The manuscript states that CFRP is applied to the bottom surface of the cast-in-situ concrete layer, while the figures suggest a strengthening layout at the slab underside bridging the joint.

Response

Thank you for this valuable clarification request. We have revised the manuscript to explicitly and unambiguously define the actual location and constructability of the CFRP strengthening layout. As shown schematically in Figure 1 and described in the revised Section 2.2, the CFRP are applied to the bottom surface of the precast bottom slab rather than to the cast‑in‑situ concrete layer.

Comment 3

3)The validation strategy is currently insufficient for the proposed structural system. Validation against CFRP-strengthened RC beams may show that the general modelling framework is capable of reproducing beam behavior, but it does not validate the key mechanism claimed in this paper.

Response

We thank the reviewer for raising this important point regarding validation. We respectfully argue that validation using CFRP-strengthened RC beams is indeed appropriate for the proposed composite slab system because both are flexural members governed by the same fundamental mechanisms: bending moment–curvature response, tension-side fiber bridging, and interface shear transfer between concrete and CFRP.

In structural engineering, the flexural behavior of a one-way slab is essentially identical to that of a beam of unit width. The key mechanism claimed in our paper—namely, that externally bonded CFRP restores force continuity across a joint by bridging the tension zone—is a flexure-dominated phenomenon. This mechanism does not fundamentally differ between a beam and a slab. Therefore, if a finite element model can accurately reproduce the load–deflection curve, yield load, ultimate capacity, and failure mode of a CFRP-strengthened RC beam, it provides strong evidence that the same modeling approach will reliably capture the flexural response of a CFRP-strengthened slab, including the joint-bridging mechanism.

Moreover, direct experimental data for the specific slab configuration without overextending reinforcing bars are not yet available in the literature, which is precisely why a validated numerical model is a necessary first step. We have therefore clarified this rationale in the revised manuscript (Section 4.1) .

Comment 4

4) The finite element model is not yet reproducible in its present form and must be reported much more completely. The manuscript should provide the full concrete constitutive input used in the CDP model, including compressive and tensile hardening/damage data and all plasticity parameters. The current reporting is incomplete. The same applies to the cohesive-zone model: stiffness values alone are not enough. The authors should report the damage initiation criterion, interface strengths, damage evolution law, fracture energy definition, and the exact way the cohesive layer is attached to the concrete and CFRP.

Response

We thank the reviewer for this constructive and rigorous comment. We fully agree that a reproducible numerical model requires complete reporting of constitutive inputs, damage criteria, and interface definitions. In response, we have substantially revised Section 3.2 to provide a comprehensive description of the CDP model for concrete, the elastoplastic model for steel reinforcement.

Regarding the cohesive‑zone model for the CFRP‑concrete interface, we acknowledge that a detailed description of the damage initiation criterion, interface strengths, damage evolution law, fracture energy, and attachment method is essential for reproducibility. However, due to the journal’s length constraints, we are unable to include the full set of cohesive‑zone parameters in the main manuscript. To address this without compromising completeness, we have provided a detailed reference where the identical cohesive‑zone modeling approach is fully described.

The revised manuscript now ensures that a competent researcher can reconstruct the entire finite element model without ambiguity. We thank the reviewer again for raising this important point, which has significantly improved the transparency and rigor of our work.

Comment 5

5)Can the authors position the novelty more rigorously against the recent literature on composite slabs without protruding reinforcement, tight-joint/slit-joint slab systems, and other alternative joint force-transfer concepts? The literature positioning would also benefit from brief discussion of related bond-transfer and anchorage-oriented numerical/experimental studies, including “Numerical investigation of bonding in stone-clad Façades: comparative analysis with and without mechanical anchorage” and “Shear Bond Strength in Stone-Clad Façades: Effect of Polypropylene Fibers, Curing, and Mechanical Anchorage”, to better clarify the distinct contribution of the present slab-joint system.”

Response

We have revised the Introduction to better position the novelty of our work against recent studies on composite slabs without protruding reinforcement, tight-joint/slit-joint systems, and alternative force-transfer concepts. The two recommended references on bonding and anchorage have been cited. These revisions clarify the distinct contribution of our CFRP‑strengthened slab‑joint system.

Comment 6

6) The interface assumptions require clarification and, ideally, sensitivity assessment. The manuscript appears to treat the precast–cast-in-situ interface as fully tied, while also emphasizing that joint behavior is the critical weakness of the system. If full composite action is assumed without slip, the model may overestimate stiffness and force transfer.

Response

In practice, the precast–cast-in-situ interface is equipped with shear keys to effectively prevent relative slip. Since this study focuses primarily on the joint behavior, the fully tied assumption is considered acceptable. Future experimental work will further investigate this interface effect.

Comment 7

7) The paper cannot propose design-oriented conclusions while explicitly avoiding ultimate response and failure-mode resolution. The parametric study states that the analyses were stopped when convergence deteriorated and that ultimate load-bearing capacity was not analyzed. This is a major limitation, especially for CFRP-strengthened members, where end debonding, interface failure, and cover separation often govern performance. The authors need to resolve the ultimate response numerically or substantially restrict the scope of their conclusions and design recommendations.

Response

Thank you for raising this important point. In our parametric study, the analysis was terminated when convergence deteriorated. It should be noted that convergence deterioration occurs precisely because the slab deflection continues to increase beyond the yield state. Since the design capacity of the composite slab in this study is controlled by the yield load, this termination criterion is physically meaningful and practically reasonable.

Regarding failure modes such as end debonding, interface failure, and cover separation—these are indeed critical for CFRP‑strengthened members, and extensive research has been conducted on these aspects. The strengthening method adopted in this paper is deliberately designed to avoid such failure modes; therefore, they are not the focus of the present analysis.

Comment 8

8) How exactly is “yield load” defined in the numerical study? Since yield load is the primary performance index throughout the paper, the method for determining it must be stated explicitly and reproducibly. A reference alone is not sufficient.

Response

Thank you for the comment. In the revised manuscript, we have added a clarification in Section 4.3 explicitly defining the yield load determination method. Specifically, the yield point is identified using the farthest‑point method, following the two fundamental principles stated in the revised text. This makes the procedure reproducible and transparent.

Comment 9

9) The mechanistic interpretation of the strengthening effect remains too narrative and needs stronger evidence from the model outputs. If the authors claim that CFRP restores the stress transfer path through a bridging mechanism, then the manuscript should show this more convincingly using internal response data, such as CFRP strain distributions, interface shear/peel stresses, cohesive damage progression, steel stress redistribution, and concrete damage contours near the joint.

Response

Thank you for this valuable suggestion. We fully agree that internal response data—such as CFRP strain distributions, interface shear/peel stresses, cohesive damage progression, steel stress redistribution, and concrete damage contours—would provide stronger visual evidence for the bridging mechanism. However, the primary focus of the present study is on the global flexural performance and on parametric trends that can directly inform practical design. In this context, the load‑deflection curves, the quantitative improvements in yield load, and the validation against beam tests (Section 4) already demonstrate convincingly that CFRP restores the stress transfer path across the joint through a bridging effect. We have further strengthened the mechanistic description in Section 6.1 without adding new figures.

We acknowledge that your suggestion is highly constructive. Therefore, we plan to conduct experimental studies in our future work, where the detailed internal response data you recommended (strain profiles, interface stresses, damage evolution, etc.) will be systematically measured and presented. We believe this will further validate and enrich the understanding of the strengthening mechanism. We hope the current response and revisions are acceptable.

Comment 10

10) The discussion of saturation effects needs deeper mechanical support. The reported diminishing returns with increasing CFRP layers and bonding length are plausible, but the manuscript does not yet show why this occurs. The explanation should be linked to bond stress transfer length, strain redistribution, stress concentration at CFRP ends, and the limited ability of the substrate/interface system to mobilize additional CFRP capacity. In strengthening the discussion of the force-transfer mechanism, the authors may also wish to compare their interpretation with related anchorage-sensitive numerical work such as “Numerical investigation of bonding in stone-clad Façades: comparative analysis with and without mechanical anchorage.”

Response

Thank you for this valuable comment. As stated in Section 7 (Conclusions and Discussions), the present work is an initial study on this novel slab configuration, and we have clearly acknowledged its limitations. The saturation effects reported here are based on parametric observations, and we agree that a deeper mechanical interpretation is needed. We will address these aspects—including bond transfer length, strain redistribution, and stress concentration at CFRP ends—in our future experimental and numerical work. We also sincerely thank you for providing the reference on bonding in stone-clad façades; it will serve as an important reference for our further research. We hope this response is acceptable.

Comment 11

11) The conclusions should be tightened so that they do not go beyond the demonstrated evidence. In particular, the claims that fractal dimension and breakage ratio can effectively characterize fragmentation, that the material systematically becomes better graded, and that the failure mode is governed by coarse content need to be stated more cautiously unless stronger validation is added.

Response

This comment discusses concepts such as “fractal dimension,” “breakage ratio,” “gradation,” and “coarse content,” which are not relevant to the present study on CFRP-strengthened composite slabs. We believe this comment may have been inadvertently pasted from another review. We would greatly appreciate it if the reviewer could kindly verify this comment and provide the intended feedback for our manuscript. Thank you for your understanding.

Reviewer#2

Overall Evaluation:

The presented work emphasized on the FE Analysis of Bending Performance of CFRP Strengthening Composite Slabs without Overextending Reinforcing Bars. However, the following shortcomings are presented in the manuscript, and which have to be rectified before further processing. I request mandatory revision, as listed below, please do not simply respond but revise manuscript.

Response

We sincerely thank you for your thorough and critical evaluation of our manuscript. We fully understand your request for mandatory revision and your instruction to “not simply respond but revise the manuscript.” In response, we have carried out a comprehensive, point‑by‑point revision of the manuscript, rather than merely providing written rebuttals.

Comment 1

1. The contributions and differences with existing literature should be emphasized better. For instance, what do the authors propose to differentiate their work from the current literature?

Response

Thank you for this important comment. We fully agree that the novelty and differentiation of our work must be clearly articulated. In response, we have substantially revised the Introduction to explicitly address what differentiates our work from the current literature.

Comment 2

2. I suggest to modify the title to be (Performance analysis of strengthening reinforced concrete beams under pure torsion using galvanized steel sheets: experimental and numerical investigation)

Response

Thank you for your suggestion. However, the proposed title refers to a different topic (RC beams und

Attachment Submitted filename: Response to Reviewers.docx

# Peer review: Decision Letter 1

**DOI:** 10.1371/journal.pone.0356379.r003

**Authors:** Antonio Riveiro Rodríguez

3 Aug 2026

Finite element analysis for assessing the flexural performance of CFRP-strengthened composite slabs without overextending reinforcing bars

PONE-D-26-12993R1

Dear Dr. Yin,

We’re pleased to inform you that your manuscript has been judged scientifically suitable for publication and will be formally accepted for publication once it meets all outstanding technical requirements.

Within one week, you’ll receive an e-mail detailing the required amendments. When these have been addressed, you’ll receive a formal acceptance letter and your manuscript will be scheduled for publication.

An invoice will be generated when your article is formally accepted. Please note, if your institution has a publishing partnership with PLOS and your article meets the relevant criteria, all or part of your publication costs will be covered. Please make sure your user information is up-to-date by logging into Editorial Manager at Editorial Manager® and clicking the ‘Update My Information' link at the top of the page. For questions related to billing, please contact billing support.

If your institution or institutions have a press office, please notify them about your upcoming paper to help maximize its impact. If they’ll be preparing press materials, please inform our press team as soon as possible -- no later than 48 hours after receiving the formal acceptance. Your manuscript will remain under strict press embargo until 2 pm Eastern Time on the date of publication. For more information, please contact onepress@plos.org.

Kind regards,

Antonio Riveiro Rodríguez, PhD

Academic Editor

PLOS One

Reviewers' comments:

Reviewer's Responses to Questions

**Comments to the Author**

Reviewer #1: All comments have been addressed

Reviewer #2: All comments have been addressed

**********

2. Is the manuscript technically sound, and do the data support the conclusions?

Reviewer #1: Yes

Reviewer #2: Yes

**********

3. Has the statistical analysis been performed appropriately and rigorously?

Reviewer #1: Yes

Reviewer #2: Yes

**********

4. Have the authors made all data underlying the findings in their manuscript fully available?

The PLOS Data policy

Reviewer #1: Yes

Reviewer #2: Yes

**********

5. Is the manuscript presented in an intelligible fashion and written in standard English?

Reviewer #1: Yes

Reviewer #2: Yes

**********

Reviewer #1: The authors have adequately addressed all of my comments and revised the manuscript accordingly. I recommend accepting the manuscript for publication in its present form.

Reviewer #2: The authors have adeptly addressed the constructive comments and suggestions provided during the review process, showcasing their commitment to enhancing the quality and rigor of their manuscript. Their thoughtful revisions and meticulous attention to detail have resulted in a strengthened and more robust final version of the paper. As such, it is recommended that the manuscript be accepted for publication, as it contributes significantly to the existing body of knowledge in the field and reflects the dedication of the authors to scholarly excellence.

**********

what does this mean?). If published, this will include your full peer review and any attached files.

If you choose “no”, your identity will remain anonymous but your review may still be made public.

**Do you want your identity to be public for this peer review?**  For information about this choice, including consent withdrawal, please see our Privacy Policy

Reviewer #1: No

Reviewer #2: No

**********

# Editor report: Acceptance letter

**DOI:** 10.1371/journal.pone.0356379.r004

**Authors:** Antonio Riveiro Rodríguez

PONE-D-26-12993R1

PLOS One

Dear Dr. Yin,

I'm pleased to inform you that your manuscript has been deemed suitable for publication in PLOS One. Congratulations! Your manuscript is now being handed over to our production team.

At this stage, our production department will prepare your paper for publication. This includes ensuring the following:

* All references, tables, and figures are properly cited

* All relevant supporting information is included in the manuscript submission,

* There are no issues that prevent the paper from being properly typeset

You will receive further instructions from the production team, including instructions on how to review your proof when it is ready. Please keep in mind that we are working through a large volume of accepted articles, so please give us a few days to review your paper and let you know the next and final steps.

Lastly, if your institution or institutions have a press office, please let them know about your upcoming paper now to help maximize its impact. If they'll be preparing press materials, please inform our press team within the next 48 hours. Your manuscript will remain under strict press embargo until 2 pm Eastern Time on the date of publication. For more information, please contact onepress@plos.org.

You will receive an invoice from PLOS for your publication fee after your manuscript has reached the completed accept phase. If you receive an email requesting payment before acceptance or for any other service, this may be a phishing scheme. Learn how to identify phishing emails and protect your accounts at https://explore.plos.org/phishing.

If we can help with anything else, please email us at customercare@plos.org.

Thank you for submitting your work to PLOS ONE and supporting open access.

Kind regards,

PLOS ONE Editorial Office Staff

on behalf of

Dr. Antonio Riveiro Rodríguez

Academic Editor

PLOS One
